# RGG: A general GUI Framework for R scripts

**DOI:** 10.1186/1471-2105-10-74

**Published:** 2009-03-02

**Authors:** Ilhami Visne, Erkan Dilaveroglu, Klemens Vierlinger, Martin Lauss, Ahmet Yildiz, Andreas Weinhaeusel, Christa Noehammer, Friedrich Leisch, Albert Kriegner

**Affiliations:** 1 Austrian Research Centers GmbH – ARC, Molecular Diagnostics, A-2444 Seibersdorf, Austria; 2 Institut für Statistik, Ludwig-Maximilians-Universität, Ludwigstraße 33, D-80539 München, Germany

## Abstract

**Background:**

R is the leading open source statistics software with a vast number of biostatistical and bioinformatical analysis packages. To exploit the advantages of R, extensive scripting/programming skills are required.

**Results:**

We have developed a software tool called R GUI Generator (RGG) which enables the easy generation of Graphical User Interfaces (GUIs) for the programming language R by adding a few Extensible Markup Language (XML) – tags. RGG consists of an XML-based GUI definition language and a Java-based GUI engine. GUIs are generated in runtime from defined GUI tags that are embedded into the R script. User-GUI input is returned to the R code and replaces the XML-tags. RGG files can be developed using any text editor. The current version of RGG is available as a stand-alone software (RGGRunner) and as a plug-in for JGR.

**Conclusion:**

RGG is a general GUI framework for R that has the potential to introduce R statistics (R packages, built-in functions and scripts) to users with limited programming skills and helps to bridge the gap between R developers and GUI-dependent users. RGG aims to abstract the GUI development from individual GUI toolkits by using an XML-based GUI definition language. Thus RGG can be easily integrated in any software. The RGG project further includes the development of a web-based repository for RGG-GUIs. RGG is an open source project licensed under the Lesser General Public License (LGPL) and can be downloaded freely at

## Background

R is an environment in which statistical techniques can be implemented [1]. R is an open source project, licensed under the General Public License, and is used by a growing number of researchers in the field of bioinformatics/biostatistics. Many new statistical procedures are published with corresponding R scripts or packages. Over 1,600 packages can be obtained from the Comprehensive R Archive Network [2] and further 300 bioinformatics packages can be obtained from the website of the Bioconductor project [3]. Notably, microarray analysis has been greatly improved by several packages like limma [4], affy [5], siggenes/samr [6], pamr [7], limmaGUI [8] and by software applications like BRB-ArrayTools [9] or by web applications like CARMAweb [10], MIDAW [11], and RACE [12] (all of these are using R and Bioconductor). R is based on an effective programming language that includes conditionals, loops, user-defined recursive functions as well as input and output facilities. The user frequently needs to change R code like the one for setting the working directory, reading an input file and changing parameters to new values, which usually requires profound knowledge of the R scripting language. For many applications, large parts of the code remain unchanged.

There are different approaches for the GUI-based usage of R: an option is the use of GUI toolkits such as Tcl/Tk [13], Gtk2 [14], Java Swing [15] and wxWidgets [16] employing the R packages tcltk [17], RGtk2 [18], rJava [19] and RwxWidgets [20]. Every toolkit has special characteristics that require the programming of its own custom GUI. gWidgets [21] serve as an abstraction of the named R packages and offers a standard gateway. A GUI that is written using gWidgets is easily compatible with these toolkits and can thus be executed. All these packages including gWidgets use R to describe GUIs and require R to generate the GUI. Such GUIs depend on R and are less flexibly integrated in other softwares.

Stand-alone frontends present another approach for the GUI-based usage of R. Rcmdr [22] and Rkward [23] runs on Linux and has a similar usability to Rcmdr yet with a richer interface. The user interface of Rkward was developed using the Qt Toolkit [24] in C++ [25]. Like in RGG, GUIs are described with XML making it easier for the end-user to expand Rkward with further GUI functions. Rpad [26] is a web-based analysis program which integrates R to an Apache web server on the backend. The R code embedded in the HTML is sent back to the server for statistical evaluation. Extending these tools to new functions requires programming skills in HTML, JavaScript [27] and/or PHP [28].

In this context, this project's aim is to develop a general GUI Framework for R. This should be independent from the GUI toolkits and integrating software and make R based statistical computing available to a wider audience less familiar with R programming.

## Implementation

The R GUI Generator (RGG) framework allows the R user to generate Graphical User Interfaces (GUIs) for R scripts. RGG uses a component-based approach and consists of two parts: an XML [29] (Extensible Markup Language)-based GUI definition language to use the existing components (GUI elements) and a GUI engine that is implemented in Java.

### RGG – GUI Engine

Within the RGG framework, the GUI engine is the interpreter of an RGG file which generates the GUI in runtime. GUI engines are language-specific and contain the implementation of GUI elements. The current engine was implemented in Java using the Java Swing Library.

However, new engines using other programming languages including R based on the same RGG GUI definition language specification may further be implemented. Thus the RGG GUI definition language is independent from the GUI toolkit.

### Java based RGG GUI engine

**Figure 1:**
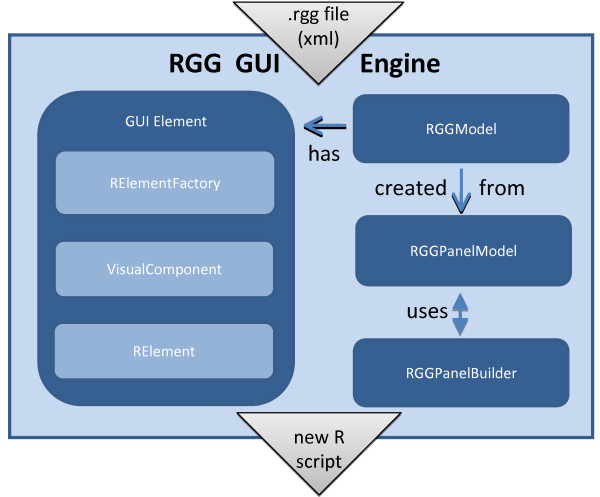
**Software Architecture of Java based RGG GUI Engine**. The GUI has four main parts: GUI elements, RGGModel, RGGPanelModel, and RGGPanelBuilder. The engine creates GUI elements for each XML tag. These GUI elements are added to the RGGModel from which the new R script is then generated. The RGGPanelModel is created from the RGGModel. RGGPanelBuilder generates the GUI using RGGPanelModel. The GUI element has three subparts: RElement, VisualComponent, and RElementFactory. RElementFactory initializes the RElement and its visual parts, the VisualComponent. VisualComponent draws the GUI elements on the screen and RElement converts the user inputs into the corresponding R code.

Figure 1 shows the software architecture of the RGG GUI engine. It consists of four parts: the GUI elements, the RGGModel, the RGGPanelModel, and the RGGPanelBuilder. The GUI engine creates the GUI elements for each XML tag in the *rgg *file. These GUI elements are added to the RGGModel from which the new R script is generated. The RGGPanelModel is created from the RGGModel and contains the GUI model. It has a two-dimensional structure for laying out the GUI widgets in rows and columns. The RGGPanelBuilder generates the GUI using the RGGPanelModel. The GUI element consists of three subcomponents, an RElement, a VisualComponent, and an RElementFactory. The RElementFactory receives XML tags including their attributes from the RGG file and initializes the RElements and their visual components. The VisualComponent draws the GUI elements on the screen and the RElement converts the user inputs into the corresponding R code.

### RGG – GUI definition Language

The RGG GUI definition language is based on XML and contains all basic GUI elements. RGG files are thus XML files. Rules applying to an XML file also apply to RGG files. To distinguish RGG files from other XML files, the root tag must be set to <rgg>. In between the two tags <rgg> and </rgg> the user can combine GUI elements and R code. There are common GUI elements such as <filechooser>, <slider>, <checkbox> or more complex and specific elements like a dataframe/matrix editor <matrix> or an importer of microarray data, the <maimporter>. Each GUI element consists of a given number of GUI widgets. Frequently, the basic elements like <textfield> (a label and a text field), <filechooser> (a label, a text field, and a button), consist of two or three GUI widgets. However, there are no limitations to the complexity of GUI elements. A file containing both R code and GUI elements is saved with the suffix .rgg. Such RGG files serve as templates for the GUI engine. The GUI engine loads the RGG file and dynamically creates and arranges GUI elements from the XML tags. Each GUI element converts the user settings (e.g. text in a <textfield>) into the corresponding R code; i.e., the XML tags in the RGG file are replaced by the corresponding R codes. In this way a new R script is generated which is then executed in R. Several XML tags leave the R script unchanged, but improve the GUI layout. These tags include the container elements <vbox> and <hbox>. The GUI components and elements can be arranged both horizontally and vertically, i.e. spread across several columns and rows of the GUI. By default, GUI components and elements are arranged vertically. To ensure horizontal arrangement of child elements, the container element <hbox> can be used, whereas the vertical arrangement can be restored using <vbox>. The <separator> tag introduces a horizontal line to separate groups of independent GUI elements, and the <gaprow> tag modifies the spatial separation of single GUI elements. Each GUI element has its attributes, which aids its fine-tuning. Some attributes are common in many GUI elements such as "var" and "label". The var attribute's value corresponds to the R variable name that the returned user input is assigned to. The label attribute defines the label of the element in the GUI. Each of the more than fifteen GUI elements and their definition by XML tags are described in detail on the project webpage  and an HTML page (provided as Additional file 1) gives an overview of the GUI elements and their attributes. New GUI elements are continuously uploaded and documented on the website. The development of RGG was inspired by XUL, several components such as the <hbox> and <vbox> concept were directly adapted from the XUL. The reason why we could not use XUL directly is related to the choice of the programming language JAVA which should make it easier to integrate RGG in other JAVA based softwares (e.g. Taverna) [30].

### JGR Plug-in & RGGRunner

**Figure 2:**
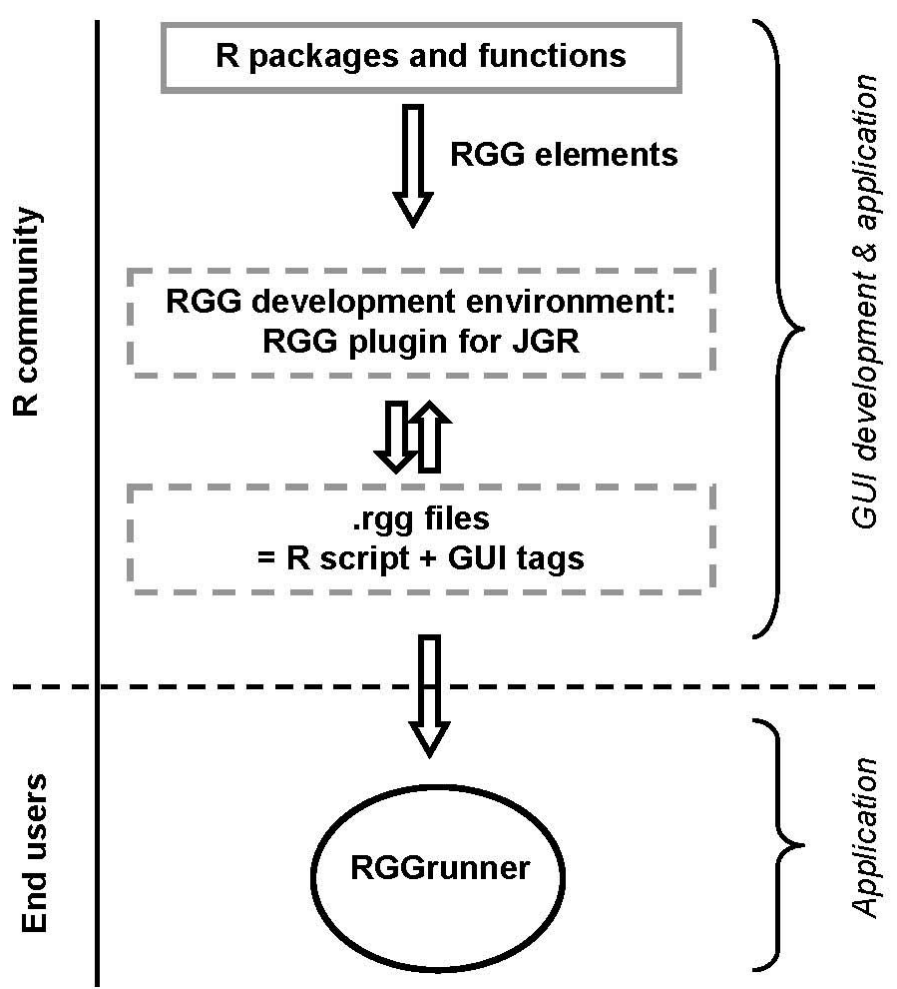
**Concept of R GUI Generator (RGG)**. For the development of R Graphical User Interfaces (GUIs) we recommend the RGG plug-in for JGR, however, any text editor can be used. Combining an R script with predefined GUI definition tags (RGG elements) results in an *rgg *file. The stand-alone software RGGRunner interprets the *rgg *file and generates the GUI for the end-user with no R skills.

The current version of RGG is available as a stand-alone software (RGGRunner) and as a plug-in for JGR (Figure 2), which is a Java frontend for R [31]. RGG generates an R script that is executed locally by RGGRunner or JGR-Plug-in. However, new execution environments can be developed, which provide a server-client architecture e.g. via Rserve. RGG does not have a server-client architecture yet, and runs R scripts locally (by JGR-Plug-in and RGGRunner). RGG files can be developed in any text editor; however, using the RGG plug-in for JGR, the developer can control both the R prompt and the nascent GUI. The RGG framework can be integrated into any software supporting R and Java. Users with limited R programming skills can operate RGG using the stand-alone application RGGRunner (provided as Additional File 2 and 3), where any given GUI can be imported and executed. Further, the source code of an RGG file and the generated R code can be viewed directly within RGGRunner. The RGGRunner is available for all major operating systems, such as Linux, Windows and Mac OS X.

### Outlook

The RGG project is going to provide a well-organized and documented web-repository for RGG files. In addition, the RGG framework will be integrated into the Taverna environment which enables the composition of bioinformatic workflows [32]. RGG is an open source project under the Lesser General Public License. The source code of these elements and of the GUI engine is freely available via a public subversion repository (see project web-site). Downloads and installation instructions are available at the project homepage .

## Simple example

**Figure 3:**
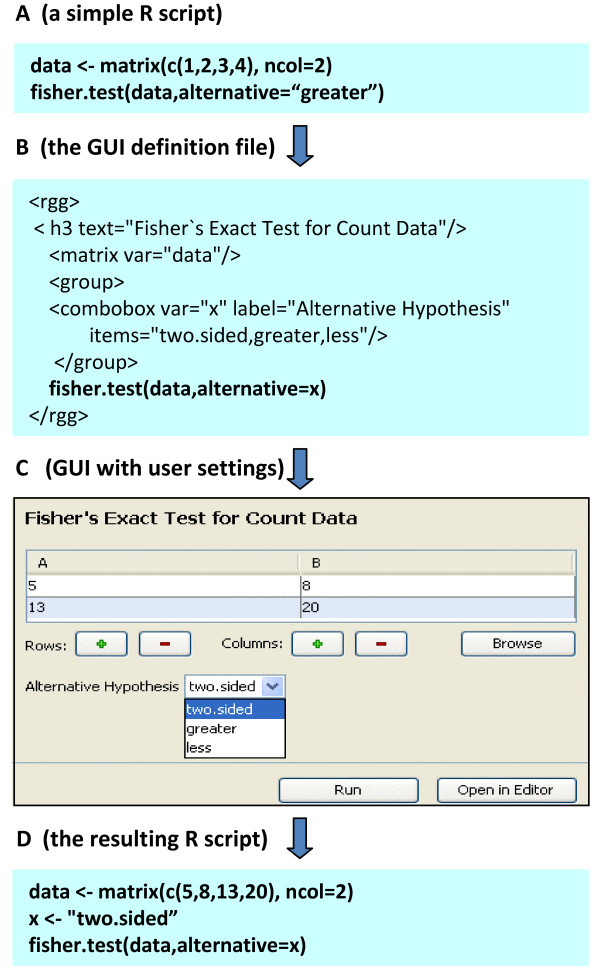
**Development of a short sample GUI using the RGG plug-in for JGR**. A) Native R script, B) *rgg *file (R script + GUI definition), C) the generated GUI, D) R commands resulting from GUI-user interaction. R code is highlighted in bold.

It may be desirable that a successful R script evolves into a GUI based function. Figure 3A gives an example of a self-explaining R-script for a Fisher's exact test on a 2 × 2 contingency table, a test frequently used in biomedical research. To run the Fisher test in R with different count data, the numeric values have to be changed in this R script. The user has to change the R script accordingly, which requires knowledge of R scripting. An RGG-GUI was created for the Fisher's exact test using a text editor or, alternatively, the RGG plug-in for JGR. This GUI includes the RGG GUI elements matrix editor <matrix>, dropdown menu <combobox> and a header text <h3 text> (Figure 3B). In the matrix editor, the numeric values can be typed in manually, or a data file can be imported by clicking on the 'Browse' button. Furthermore, rows and columns can be added or deleted from the matrix. In the additional drop down menu the test may be chosen to be 'two-sided' (Figure 3C). The resulting R code (Figure 3D), that is returned from the user-GUI input, is finally executed in R by clicking the 'Run' button. The GUI element 'matrix editor' can be restricted to two rows/columns. Alternatively, larger matrices can be allowed when the according function parameters are also supported by appropriate GUI elements in the RGG file. For a generic RGG for the Fisher's exact test, see Additional File 4. To obtain a user-friendly GUI for more sophisticated RGG files, the arrangement of the GUI elements and its components should be carefully planned in advance. The following procedure for the development of an RGG file is recommended: first, the GUI elements have to be selected. Second, the GUI elements are arranged horizontally and vertically on a virtual grid (using the <hbox> and <vbox> elements), keeping the component composition of each element in mind. Third, the selected GUI elements have to be put in their correct positions in the R script. More sample RGG files are available at the project homepage.

## Results and discussion

We have developed a general GUI framework for R scripts for a broad range of R users: Developers with skills in XML and R can provide GUIs, whereas users of bioinformatics software (without programming skills) can run those GUIs with the software RGGRunner.

RGG consists of an XML based GUI definition language and a GUI engine that interprets the GUI language and generates a new R code. Describing GUIs with XML decouples RGG from any concrete GUI toolkit. Unlike R based toolkits such as gWidgets [21], XML simplifies the integration of RGG in software not developed in R. The current version of RGG is available as stand-alone software RGGRunner and as a plug-in for JGR.

### RGG-GUI application

**Figure 4:**
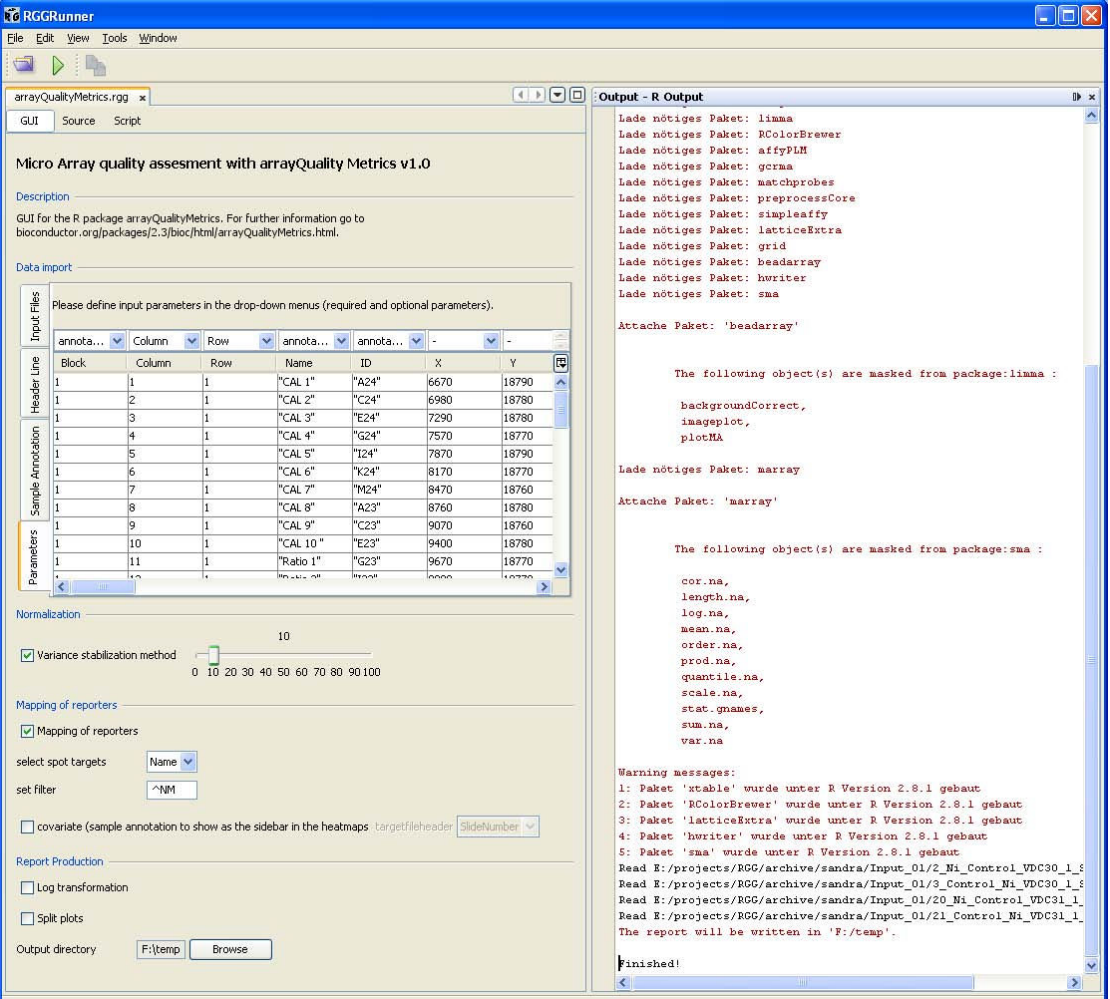
**An *rgg *file for the package 'arrayQualityMetrics' executed with the stand-alone software RGGRunner**. The GUI consists of several GUI elements: a microarray importer, a checkbox and a slider for 'vsn normalization', a checkbox and further elements for 'mapping of reporters', two checkboxes for report production parameters and a filechooser. The output log from the R console resulting from the GUI-user interaction is returned to the window on the right. An output folder is created containing the results/plots recorded by the 'arrayQualityMetrics' package.

To execute RGG files, they are loaded into the RGGRunner software (for requirements and installation instructions see the project homepage). The current version of RGGRunner doesn't include a graphic device yet. Therefore the plots are automatically saved in the working directory. Figure 3 illustrates a simple example. However, following this concept, any statistical function, including more complex scripts and packages, can be made available for the RGG framework. Routine analysis of complex data, like data obtained from microarray analysis, is an important bioinformatical application field of R. As a starting point for analysis, microarray raw data files, have to be imported using the corresponding R package for the microarray platform. Then the data are pre-processed and the quality of the microarray data is assessed. In Figure 4 we demonstrate an RGG-GUI for the R package 'arrayQualityMetrics', suitable for the assessment of microarray quality by experimentators with limited R programming skills. The RGGRunner interprets an RGG file that contains the GUI element micorarray importer <maimporter>, and downstream GUI elements for the function parameters of the 'arrayQualityMetrics' package (Additional File 5). These downstream elements include two checkboxes for normalization and mapping of reporters, respectively, which enable further GUI elements when checked by the GUI user. Finally there are two checkboxes for report production options and a file-chooser to set the output directory for the results.

The GUI element 'microarray importer' generalizes the import of microarray files by using a wizard system. The importer reads the raw data files and/or the experiment descriptor file and then automatically detects the microarray platform: If the raw data files are Affymetrix ('CEL') files, then the 'readAffy' function from the 'affy' package [5] is used and an 'AffyBatch'-object is generated. In the case of Agilent, GenePix, or other custom array data files, an 'RGlist' object is generated by the 'read.maimages' function of the 'limma' package [4]. The user-interaction for the downstream GUI elements (an optional 'vsn' normalization procedure from the 'arrayQualityMetrics' package, an optional mapping of reporters, and the setting of parameters of the main function) is further interpreted. The resulting R commands can be checked in the log on the right window of the RGGRunner. All output from the quality control package 'arrayQualityMetrics' is saved to the selected project folder. This GUI can be extended by customized downstream analysis steps for the entire analysis of micorarray data, including subsequent pathway [33] and gene-ontology [34] analysis.

### Perspectives

One of the biggest advantages of R is its large and active user community, providing a vast number of scripts, functions and packages. Through a common GUI definition language for R, the generation of R GUIs could evolve into a community effort. Each standard statistical function such as built-in functions (e.g. t-test, ANOVA) and packages (Bioconductor, CRAN, etc.) could be provided with standard GUIs. A unified concept of GUI generation would also facilitate the teaching of introductory as well as of advanced statistics lectures. In the course of the rapid methodological progress in life sciences, novel (bio)statistical approaches are permanently required. When provided on a GUI base, novel statistical procedures can be shared directly with other GUI-dependent users. The R package 'Sweave' documents R analysis by integrating R commands and output text, figures and tables into a pdf/LaTeX report [35] and thus could contribute to the transparency of research findings [36]. If used together with RGG, pdf/LaTeX files could be produced much faster.

RGG is based on an intuitive GUI definition language which enables the description of GUIs declaratively, without extensive programming, directly by R users. RGG provides a GUI framework that is designed to be integrated with different R frontends and other softwares supporting R. Currently the RGG project is at the stage of a general framework but does not provide a collection of GUIs. At the moment, our focus is on writing RGG files for the standard functions of R and to extend the range of available GUI elements. However, RGG files for complex and specific statistical procedures can only be efficiently developed by the R community. Therefore, the community is invited to take advantage of the RGG framework and to submit biostatistical solutions not only as R packages/scripts but also with concomitant RGG files. Clearly, the addition of such RGG libraries, would significantly contribute to RGG's utility in many scientific studies.

## Conclusion

We introduce a generic concept that allows R users to extend their statistical and bioinformatical procedures, which are relying on commands in the R prompt, with standard graphical user interfaces. The R script developer can use the RGG framework to make biostatistical and bioinformatical solutions directly available for routine applications. The current project will further include the development of a web repository for RGG files and integration to a workflow environment, enabling the combination of GUIs and connection to web services and databases. Such community driven collections of RGG-GUIs and analytical pipelines could help to bridge the gap between the R developer community and GUI-dependent users with limited R scripting skills.

## Availability and requirements

**Project name: **RGG

**Project home page: **

**Operating system(s): **All major platforms, such as Linux, Windows and Mac OS X

**Other requirements: **R 2.8.x or newer, Java 5 or newer, JGR 1.6 or newer is recommended

**License: **LGPL

**Any restrictions to use by non-academics: **LGPL

## Authors' contributions

AK and FL conceived of the project, designed the software/framework and supervised software implementation. IV programmed the RGG GUI engine and the project's web-site. AY implemented the microarray importer GUI element. ED implemented the RGGRunner. KV and AW implemented sample RGG GUIs and provided R test scripts. CN was responsible for software testing and defined conceptual requirements. ML wrote the manuscript and was additionally involved in software development. All authors read and approved the manuscript.

## Supplementary Material

Additional file 1 **Supplementary table**. Table of RGG GUI Elements and Their Attributes. Click here for file

Additional file 2 **RGG runner for Windows/Linux**. Software to load and execute *rgg *files (Windows and Linux). Click here for file

Additional file 3 **RGG runner for**. Software to load and execute *rgg *files (Mac OS X). Mac OS X. Click here for file

Additional file 4 **Fisher Test**. *rgg *file and sample data (to be used with RGGRunner from Additional file 2 and 3). Click here for file

Additional file 5 **Array Quality Metrics**. *rgg *file and sample data (to be used with RGGRunner from Additional file 2 and 3). Requires R version 2.7.0 or higher. A minimum of two microarray raw data files are needed to run the R package 'arrayQualityMetrics'. Click here for file

## References

[B1] R Development Core Team (2005). R: A Language and Environment for Statistical Computing.

[B2] The Comprehensive R Archive Network. http://cran.r-project.org/.

[B3] Gentleman RC, Carey VJ, Bates DM, Bolstad B, Dettling M, Dudoit S, Ellis B, Gautier L, Ge Y, Gentry J, Hornik K, Hothorn T, Huber W, Iacus S, Irizarry R, Leisch F, Li C, Maechler M, Rossini AJ, Sawitzki G, Smith C, Smyth G, Tierney L, Yang JY, Zhang J (2004). Bioconductor: open software development for computational biology and bioinformatics. Genome Biol.

[B4] Smyth GK (2004). Linear models and empirical bayes methods for assessing differential expression in microarray experiments. Stat Appl Genet Mol Biol.

[B5] Gautier L, Cope L, Bolstad BM, Irizarry RA (2004). affy – analysis of Affymetrix GeneChip data at the probe level. Bioinformatics.

[B6] Tusher VG, Tibshirani R, Chu G (2001). Significance analysis of microarrays applied to the ionizing radiation response. Proc Natl Acad Sci USA.

[B7] Tibshirani R, Hastie T, Narasimhan B, Chu G (2002). Diagnosis of multiple cancer types by shrunken centroids of gene expression. Proc Natl Acad Sci USA.

[B8] Wettenhall JM, Smyth GK (2004). limmaGUI: a graphical user interface for linear modeling of microarray data. Bioinformatics.

[B9] Xu X, Zhao Y, Simon R (2008). Gene Set Expression Comparison kit for BRB-ArrayTools. Bioinformatics.

[B10] Rainer J, Sanchez-Cabo F, Stocker G, Sturn A, Trajanoski Z (2006). CARMAweb: comprehensive R- and bioconductor-based web service for microarray data analysis. Nucleic Acids Res.

[B11] Romualdi C, Vitulo N, Del FM, Lanfranchi G (2005). MIDAW: a web tool for statistical analysis of microarray data. Nucleic Acids Res.

[B12] Psarros M, Heber S, Sick M, Thoppae G, Harshman K, Sick B (2005). RACE: Remote Analysis Computation for gene Expression data. Nucleic Acids Res.

[B13] Tcl/Tk. http://www.tcl.tk.

[B14] GTK2. http://www.gtk.org.

[B15] Java Swing. http://java.sun.com/docs/books/tutorial/uiswing.

[B16] wxWidgets. http://wxwidgets.org.

[B17] Dalgaard P, Hornik K, Leisch F The R-Tcl/Tk Interface. Proceedings of the 2nd International Workshop on Distributed Statistical Computing: March 15–17 2001; Vienna.

[B18] RGtk2. http://www.ggobi.org/rgtk2.

[B19] rJava. http://www.rforge.net/rJava.

[B20] RwxWidgets. http://www.omegahat.org/RwxWidgets.

[B21] gWidgets. http://wiener.math.csi.cuny.edu/pmg/gWidgets.

[B22] Fox J (2005). Rcmdr: The R Commander: A Basic-Statistics Graphical User Interface to R. Journalof Statistical Software.

[B23] Rkward. http://rkward.sourceforge.net/.

[B24] Qt Toolkit. http://trolltech.com/products.

[B25] C++. http://en.wikipedia.org/wiki/C%2B%2B.

[B26] Rpad. http://www.rpad.org/Rpad/.

[B27] JavaScript. http://www.mozilla.org/rhino/.

[B28] PHP. http://www.php.net/.

[B29] Extensible Markup Language. http://www.w3.org/XML/.

[B30] XML User Interface Language. http://www.mozilla.org/projects/xul/.

[B31] Helbig M, Theus M, Urbanek S (2005). JGR: Java GUI for R. Statistical Computing and Graphics Newsletter.

[B32] Oinn T, Addis M, Ferris J, Marvin D, Senger M, Greenwood M, Carver T, Glover K, Pocock MR, Wipat A, Li P (2004). Taverna: a tool for the composition and enactment of bioinformatics workflows. Bioinformatics.

[B33] Kanehisa M, Goto S, Hattori M, Aoki-Kinoshita KF, Itoh M, Kawashima S, Katayama T, Araki M, Hirakawa M (2006). From genomics to chemical genomics: new developments in KEGG. Nucleic Acids Res.

[B34] Ashburner M, Ball CA, Blake JA, Botstein D, Butler H, Cherry JM, Davis AP, Dolinski K, Dwight SS, Eppig JT, Harris MA, Hill DP, Issel-Tarver L, Kasarskis A, Lewis S, Matese JC, Richardson JE, Ringwald M, Rubin GM, Sherlock G (2000). Gene ontology: tool for the unification of biology. The Gene Ontology Consortium. Nat Genet.

[B35] Leisch F, Härdle W, Rönz B (2002). Sweave: dynamic generation of statistical reports using literate data analysis. Proceedings in Computational Statistics.

[B36] Ioannidis JP, Polyzos NP, Trikalinos TA (2007). Selective discussion and transparency in microarray research findings for cancer outcomes. Eur J Cancer.

