# Supplementary material for: RGG: A general GUI Framework for R scripts
Source: BMC Bioinformatics. 2009 Mar 2;10:74. doi: 10.1186/1471-2105-10-74 (PMC2653488; doi:10.1186/1471-2105-10-74)
Supplement: Additional file 1 — Supplementary table. Table of RGG GUI Elements and Their Attributes. [file 1471-2105-10-74-S1.html]

Table of RGG GUI Elements and Their Attributes


## Table of RGG GUI Elements and Their Attributes

Basic GUI Elements

| GUI Element | Attributes |
| --- | --- |
| filechooser | var, label, accepted-extension, fileselection-mode, multiselection-enabled, description, span |
| textfield | var, label, data-type, default-value, size, span |
| combobox | var, label, items, selected-item, selected-index, span |
| slider | var, label, min, max, default-value, paint-ticks, paint-labels, paint-track, paint-value, major-tick-spacing, minor-tick-spacing, span |
| checkbox | var, label, selected, return-value-by-selected, return-value-by-notselected, labelposition, span |
| radiobutton | var, label, button-group, selected, return-value-by-selected, labelposition, span |
| listbox | var, label, visible-row-count, data-type, span |
| vector | var, label, vector-type, size, default-value, alignment |
| matrix | var, data-type, alignment, span |
| vbox |  |
| hbox |  |
| label | test, alignment, span |
| h1 | text, span, alignment |
| h2 | text, span, alignment |
| h3 | text, span, alignment |
| h4 | text, span, alignment |
| h5 | text, span, alignment |
| h6 | text, span, alignment |
| img | src, alignment, span |
| separator | label, span |
| gaprow | height |

  
Area specific GUI Elements  
  
Micro Array Analysis  

|  |  |
| --- | --- |
| GUI Element | Attributes |
| maimporter | var, other-columns, span |
| targetfileeditor | var, span |
