# Supplementary material for: RGG: A general GUI Framework for R scripts
Source: BMC Bioinformatics. 2009 Mar 2;10:74. doi: 10.1186/1471-2105-10-74 (PMC2653488; doi:10.1186/1471-2105-10-74)
Supplement: Additional file 2 — RGG runner for Windows/Linux. Software to load and execute rgg files (Windows and Linux). [file 1471-2105-10-74-S2.zip › rggrunner/platform8/docs/swing-layout-1.0.3-doc.zip!/constant-values.html]

Constant Field Values


|  |  |  |
| --- | --- | --- |
| **Package** \| Class \| Use \| **Tree** \| **Deprecated** \| **Index** \| **Help** | |  |
| PREV   NEXT | **FRAMES**    **NO FRAMES**     **All Classes** |  |


---


# Constant Field Values


---

**Contents**

- org.jdesktop.\*

org.jdesktop.\*

| org.jdesktop.layout.Baseline | | |
| --- | --- | --- |
| `public static final int` | `BRB_CENTER_OFFSET` | `3` |
| `public static final int` | `BRB_CONSTANT_ASCENT` | `1` |
| `public static final int` | `BRB_CONSTANT_DESCENT` | `2` |
| `public static final int` | `BRB_OTHER` | `4` |

| org.jdesktop.layout.GroupLayout | | |
| --- | --- | --- |
| `public static final int` | `BASELINE` | `3` |
| `public static final int` | `CENTER` | `4` |
| `public static final int` | `DEFAULT_SIZE` | `-1` |
| `public static final int` | `HORIZONTAL` | `1` |
| `public static final int` | `LEADING` | `1` |
| `public static final int` | `PREFERRED_SIZE` | `-2` |
| `public static final int` | `TRAILING` | `2` |
| `public static final int` | `VERTICAL` | `2` |

| org.jdesktop.layout.LayoutStyle | | |
| --- | --- | --- |
| `public static final int` | `INDENT` | `3` |
| `public static final int` | `RELATED` | `0` |
| `public static final int` | `UNRELATED` | `1` |

---


|  |  |  |
| --- | --- | --- |
| **Package** \| Class \| Use \| **Tree** \| **Deprecated** \| **Index** \| **Help** | |  |
| PREV   NEXT | **FRAMES**    **NO FRAMES**     **All Classes** |  |


---
