# Supplementary material for: RGG: A general GUI Framework for R scripts
Source: BMC Bioinformatics. 2009 Mar 2;10:74. doi: 10.1186/1471-2105-10-74 (PMC2653488; doi:10.1186/1471-2105-10-74)
Supplement: Additional file 2 — RGG runner for Windows/Linux. Software to load and execute rgg files (Windows and Linux). [file 1471-2105-10-74-S2.zip › rggrunner/platform8/docs/swing-layout-1.0.3-doc.zip!/index-files/index-1.html]

A-Index


|  |  |  |
| --- | --- | --- |
| **Package** \| Class \| Use \| **Tree** \| **Deprecated** \| **Index** \| **Help** | |  |
| PREV LETTER   **NEXT LETTER** | **FRAMES**    **NO FRAMES**     **All Classes** |  |


A B C D G H I L M O P R S T U V 

---


## **A**

**add(GroupLayout.Group)** - Method in class org.jdesktop.layout.GroupLayout.ParallelGroup: Adds the specified `Group`. **add(Component)** - Method in class org.jdesktop.layout.GroupLayout.ParallelGroup: Adds the specified Component. **add(Component, int, int, int)** - Method in class org.jdesktop.layout.GroupLayout.ParallelGroup: Adds the specified `Component`. **add(int)** - Method in class org.jdesktop.layout.GroupLayout.ParallelGroup: Adds a rigid gap. **add(int, int, int)** - Method in class org.jdesktop.layout.GroupLayout.ParallelGroup: Adds a gap with the specified size. **add(int, GroupLayout.Group)** - Method in class org.jdesktop.layout.GroupLayout.ParallelGroup: Adds the specified `Group` as a child of this group. **add(int, Component)** - Method in class org.jdesktop.layout.GroupLayout.ParallelGroup: Adds the specified Component. **add(int, Component, int, int, int)** - Method in class org.jdesktop.layout.GroupLayout.ParallelGroup: Adds the specified `Component`. **add(GroupLayout.Group)** - Method in class org.jdesktop.layout.GroupLayout.ParallelGroup: Adds the specified `Group`. **add(Component)** - Method in class org.jdesktop.layout.GroupLayout.ParallelGroup: Adds the specified Component. **add(Component, int, int, int)** - Method in class org.jdesktop.layout.GroupLayout.ParallelGroup: Adds the specified `Component`. **add(int)** - Method in class org.jdesktop.layout.GroupLayout.ParallelGroup: Adds a rigid gap. **add(int, int, int)** - Method in class org.jdesktop.layout.GroupLayout.ParallelGroup: Adds a gap with the specified size. **add(int, GroupLayout.Group)** - Method in class org.jdesktop.layout.GroupLayout.ParallelGroup: Adds the specified `Group` as a child of this group. **add(int, Component)** - Method in class org.jdesktop.layout.GroupLayout.ParallelGroup: Adds the specified Component. **add(int, Component, int, int, int)** - Method in class org.jdesktop.layout.GroupLayout.ParallelGroup: Adds the specified `Component`. **add(GroupLayout.Group)** - Method in class org.jdesktop.layout.GroupLayout.SequentialGroup: Adds the specified `Group` to this `SequentialGroup` **add(boolean, GroupLayout.Group)** - Method in class org.jdesktop.layout.GroupLayout.SequentialGroup: Adds a `Group` to this `Group`. **add(Component)** - Method in class org.jdesktop.layout.GroupLayout.SequentialGroup: Adds the specified Component. **add(boolean, Component)** - Method in class org.jdesktop.layout.GroupLayout.SequentialGroup: Adds a `Component` to this `Group`. **add(Component, int, int, int)** - Method in class org.jdesktop.layout.GroupLayout.SequentialGroup: Adds the specified `Component`. **add(boolean, Component, int, int, int)** - Method in class org.jdesktop.layout.GroupLayout.SequentialGroup: Adds a `Component` to this `Group` with the specified size. **add(int)** - Method in class org.jdesktop.layout.GroupLayout.SequentialGroup: Adds a rigid gap. **add(int, int, int)** - Method in class org.jdesktop.layout.GroupLayout.SequentialGroup: Adds a gap with the specified size. **add(GroupLayout.Group)** - Method in class org.jdesktop.layout.GroupLayout.SequentialGroup: Adds the specified `Group` to this `SequentialGroup` **add(boolean, GroupLayout.Group)** - Method in class org.jdesktop.layout.GroupLayout.SequentialGroup: Adds a `Group` to this `Group`. **add(Component)** - Method in class org.jdesktop.layout.GroupLayout.SequentialGroup: Adds the specified Component. **add(boolean, Component)** - Method in class org.jdesktop.layout.GroupLayout.SequentialGroup: Adds a `Component` to this `Group`. **add(Component, int, int, int)** - Method in class org.jdesktop.layout.GroupLayout.SequentialGroup: Adds the specified `Component`. **add(boolean, Component, int, int, int)** - Method in class org.jdesktop.layout.GroupLayout.SequentialGroup: Adds a `Component` to this `Group` with the specified size. **add(int)** - Method in class org.jdesktop.layout.GroupLayout.SequentialGroup: Adds a rigid gap. **add(int, int, int)** - Method in class org.jdesktop.layout.GroupLayout.SequentialGroup: Adds a gap with the specified size. **addContainerGap()** - Method in class org.jdesktop.layout.GroupLayout.SequentialGroup: Adds an element representing the preferred gap between one edge of the container and the next/previous Component. **addContainerGap(int, int)** - Method in class org.jdesktop.layout.GroupLayout.SequentialGroup: Adds an element representing the preferred gap between one edge of the container and the next/previous Component. **addContainerGap()** - Method in class org.jdesktop.layout.GroupLayout.SequentialGroup: Adds an element representing the preferred gap between one edge of the container and the next/previous Component. **addContainerGap(int, int)** - Method in class org.jdesktop.layout.GroupLayout.SequentialGroup: Adds an element representing the preferred gap between one edge of the container and the next/previous Component. **addLayoutComponent(String, Component)** - Method in class org.jdesktop.layout.GroupLayout: Notification that a `Component` has been added to the parent container. **addLayoutComponent(Component, Object)** - Method in class org.jdesktop.layout.GroupLayout: Notification that a `Component` has been added to the parent container. **addLayoutComponent(String, Component)** - Method in class org.jdesktop.layout.GroupLayout: Notification that a `Component` has been added to the parent container. **addLayoutComponent(Component, Object)** - Method in class org.jdesktop.layout.GroupLayout: Notification that a `Component` has been added to the parent container. **addPreferredGap(JComponent, JComponent, int)** - Method in class org.jdesktop.layout.GroupLayout.SequentialGroup: Adds an element representing the preferred gap between the two components. **addPreferredGap(JComponent, JComponent, int, boolean)** - Method in class org.jdesktop.layout.GroupLayout.SequentialGroup: Adds an element representing the preferred gap between the two components. **addPreferredGap(int)** - Method in class org.jdesktop.layout.GroupLayout.SequentialGroup: Adds an element representing the preferred gap between the nearest components. **addPreferredGap(int, int, int)** - Method in class org.jdesktop.layout.GroupLayout.SequentialGroup: Adds an element for the preferred gap between the nearest components. **addPreferredGap(JComponent, JComponent, int)** - Method in class org.jdesktop.layout.GroupLayout.SequentialGroup: Adds an element representing the preferred gap between the two components. **addPreferredGap(JComponent, JComponent, int, boolean)** - Method in class org.jdesktop.layout.GroupLayout.SequentialGroup: Adds an element representing the preferred gap between the two components. **addPreferredGap(int)** - Method in class org.jdesktop.layout.GroupLayout.SequentialGroup: Adds an element representing the preferred gap between the nearest components. **addPreferredGap(int, int, int)** - Method in class org.jdesktop.layout.GroupLayout.SequentialGroup: Adds an element for the preferred gap between the nearest components.

---


|  |  |  |
| --- | --- | --- |
| **Package** \| Class \| Use \| **Tree** \| **Deprecated** \| **Index** \| **Help** | |  |
| PREV LETTER   **NEXT LETTER** | **FRAMES**    **NO FRAMES**     **All Classes** |  |


A B C D G H I L M O P R S T U V 

---
