# Supplementary material for: RGG: A general GUI Framework for R scripts
Source: BMC Bioinformatics. 2009 Mar 2;10:74. doi: 10.1186/1471-2105-10-74 (PMC2653488; doi:10.1186/1471-2105-10-74)
Supplement: Additional file 2 — RGG runner for Windows/Linux. Software to load and execute rgg files (Windows and Linux). [file 1471-2105-10-74-S2.zip › rggrunner/platform8/docs/swing-layout-1.0.3-doc.zip!/index-files/index-13.html]

S-Index


|  |  |  |
| --- | --- | --- |
| **Package** \| Class \| Use \| **Tree** \| **Deprecated** \| **Index** \| **Help** | |  |
| **PREV LETTER**   **NEXT LETTER** | **FRAMES**    **NO FRAMES**     **All Classes** |  |


A B C D G H I L M O P R S T U V 

---


## **S**

**setAutocreateContainerGaps(boolean)** - Method in class org.jdesktop.layout.GroupLayout: Sets whether or not gaps between the container and the first/last components should automatically be created. **setAutocreateContainerGaps(boolean)** - Method in class org.jdesktop.layout.GroupLayout: Sets whether or not gaps between the container and the first/last components should automatically be created. **setAutocreateGaps(boolean)** - Method in class org.jdesktop.layout.GroupLayout: Sets whether or not a gap between components should automatically be created. **setAutocreateGaps(boolean)** - Method in class org.jdesktop.layout.GroupLayout: Sets whether or not a gap between components should automatically be created. **setHonorsVisibility(boolean)** - Method in class org.jdesktop.layout.GroupLayout: Sets whether component visiblity is considered when sizing and positioning components. **setHonorsVisibility(Component, Boolean)** - Method in class org.jdesktop.layout.GroupLayout: Sets whether the component's visiblity is considered for sizing and positioning. **setHonorsVisibility(boolean)** - Method in class org.jdesktop.layout.GroupLayout: Sets whether component visiblity is considered when sizing and positioning components. **setHonorsVisibility(Component, Boolean)** - Method in class org.jdesktop.layout.GroupLayout: Sets whether the component's visiblity is considered for sizing and positioning. **setHorizontalGroup(GroupLayout.Group)** - Method in class org.jdesktop.layout.GroupLayout: Sets the `Group` that is responsible for layout along the horizontal axis. **setHorizontalGroup(GroupLayout.Group)** - Method in class org.jdesktop.layout.GroupLayout: Sets the `Group` that is responsible for layout along the horizontal axis. **setLayoutStyle(LayoutStyle)** - Method in class org.jdesktop.layout.GroupLayout: Sets the LayoutStyle this GroupLayout is to use. **setLayoutStyle(LayoutStyle)** - Method in class org.jdesktop.layout.GroupLayout: Sets the LayoutStyle this GroupLayout is to use. **setSharedInstance(LayoutStyle)** - Static method in class org.jdesktop.layout.LayoutStyle: Sets the LayoutStyle instance to use for this look and feel. **setSharedInstance(LayoutStyle)** - Static method in class org.jdesktop.layout.LayoutStyle: Sets the LayoutStyle instance to use for this look and feel. **setVerticalGroup(GroupLayout.Group)** - Method in class org.jdesktop.layout.GroupLayout: Sets the `Group` that is responsible for layout along the vertical axis. **setVerticalGroup(GroupLayout.Group)** - Method in class org.jdesktop.layout.GroupLayout: Sets the `Group` that is responsible for layout along the vertical axis.

---


|  |  |  |
| --- | --- | --- |
| **Package** \| Class \| Use \| **Tree** \| **Deprecated** \| **Index** \| **Help** | |  |
| **PREV LETTER**   **NEXT LETTER** | **FRAMES**    **NO FRAMES**     **All Classes** |  |


A B C D G H I L M O P R S T U V 

---
