# Supplementary material for: RGG: A general GUI Framework for R scripts
Source: BMC Bioinformatics. 2009 Mar 2;10:74. doi: 10.1186/1471-2105-10-74 (PMC2653488; doi:10.1186/1471-2105-10-74)
Supplement: Additional file 2 — RGG runner for Windows/Linux. Software to load and execute rgg files (Windows and Linux). [file 1471-2105-10-74-S2.zip › rggrunner/platform8/docs/swing-layout-1.0.3-doc.zip!/index-files/index-2.html]

B-Index


|  |  |  |
| --- | --- | --- |
| **Package** \| Class \| Use \| **Tree** \| **Deprecated** \| **Index** \| **Help** | |  |
| **PREV LETTER**   **NEXT LETTER** | **FRAMES**    **NO FRAMES**     **All Classes** |  |


A B C D G H I L M O P R S T U V 

---


## **B**

**Baseline** - Class in org.jdesktop.layout: Convenience class that can be used to determine the baseline of a particular component. **Baseline** - Class in org.jdesktop.layout: Convenience class that can be used to determine the baseline of a particular component. **Baseline()** - Constructor for class org.jdesktop.layout.Baseline: Creates an instance of Baseline. **Baseline()** - Constructor for class org.jdesktop.layout.Baseline: Creates an instance of Baseline. **BASELINE** - Static variable in class org.jdesktop.layout.GroupLayout: Possible alignment type. **BASELINE** - Static variable in class org.jdesktop.layout.GroupLayout: Possible alignment type. **BRB\_CENTER\_OFFSET** - Static variable in class org.jdesktop.layout.Baseline: Baseline resize behavior constant. **BRB\_CENTER\_OFFSET** - Static variable in class org.jdesktop.layout.Baseline: Baseline resize behavior constant. **BRB\_CONSTANT\_ASCENT** - Static variable in class org.jdesktop.layout.Baseline: Baseline resize behavior constant. **BRB\_CONSTANT\_ASCENT** - Static variable in class org.jdesktop.layout.Baseline: Baseline resize behavior constant. **BRB\_CONSTANT\_DESCENT** - Static variable in class org.jdesktop.layout.Baseline: Baseline resize behavior constant. **BRB\_CONSTANT\_DESCENT** - Static variable in class org.jdesktop.layout.Baseline: Baseline resize behavior constant. **BRB\_OTHER** - Static variable in class org.jdesktop.layout.Baseline: Baseline resize behavior constant. **BRB\_OTHER** - Static variable in class org.jdesktop.layout.Baseline: Baseline resize behavior constant.

---


|  |  |  |
| --- | --- | --- |
| **Package** \| Class \| Use \| **Tree** \| **Deprecated** \| **Index** \| **Help** | |  |
| **PREV LETTER**   **NEXT LETTER** | **FRAMES**    **NO FRAMES**     **All Classes** |  |


A B C D G H I L M O P R S T U V 

---
