# Supplementary material for: RGG: A general GUI Framework for R scripts
Source: BMC Bioinformatics. 2009 Mar 2;10:74. doi: 10.1186/1471-2105-10-74 (PMC2653488; doi:10.1186/1471-2105-10-74)
Supplement: Additional file 2 — RGG runner for Windows/Linux. Software to load and execute rgg files (Windows and Linux). [file 1471-2105-10-74-S2.zip › rggrunner/platform8/docs/swing-layout-1.0.3-doc.zip!/index-files/index-3.html]

C-Index


|  |  |  |
| --- | --- | --- |
| **Package** \| Class \| Use \| **Tree** \| **Deprecated** \| **Index** \| **Help** | |  |
| **PREV LETTER**   **NEXT LETTER** | **FRAMES**    **NO FRAMES**     **All Classes** |  |


A B C D G H I L M O P R S T U V 

---


## **C**

**CENTER** - Static variable in class org.jdesktop.layout.GroupLayout: Possible alignment type. **CENTER** - Static variable in class org.jdesktop.layout.GroupLayout: Possible alignment type. **createBaselineGroup(boolean, boolean)** - Method in class org.jdesktop.layout.GroupLayout: Creates and returns a `ParallelGroup` that aligns it's elements along the baseline. **createBaselineGroup(boolean, boolean)** - Method in class org.jdesktop.layout.GroupLayout: Creates and returns a `ParallelGroup` that aligns it's elements along the baseline. **createParallelGroup()** - Method in class org.jdesktop.layout.GroupLayout: Creates and returns a `ParallelGroup` with a `LEADING` alignment. **createParallelGroup(int)** - Method in class org.jdesktop.layout.GroupLayout: Creates and returns an `ParallelGroup`. **createParallelGroup(int, boolean)** - Method in class org.jdesktop.layout.GroupLayout: Creates and returns an `ParallelGroup`. **createParallelGroup()** - Method in class org.jdesktop.layout.GroupLayout: Creates and returns a `ParallelGroup` with a `LEADING` alignment. **createParallelGroup(int)** - Method in class org.jdesktop.layout.GroupLayout: Creates and returns an `ParallelGroup`. **createParallelGroup(int, boolean)** - Method in class org.jdesktop.layout.GroupLayout: Creates and returns an `ParallelGroup`. **createSequentialGroup()** - Method in class org.jdesktop.layout.GroupLayout: Creates and returns a `SequentialGroup`. **createSequentialGroup()** - Method in class org.jdesktop.layout.GroupLayout: Creates and returns a `SequentialGroup`.

---


|  |  |  |
| --- | --- | --- |
| **Package** \| Class \| Use \| **Tree** \| **Deprecated** \| **Index** \| **Help** | |  |
| **PREV LETTER**   **NEXT LETTER** | **FRAMES**    **NO FRAMES**     **All Classes** |  |


A B C D G H I L M O P R S T U V 

---
