# Supplementary material for: RGG: A general GUI Framework for R scripts
Source: BMC Bioinformatics. 2009 Mar 2;10:74. doi: 10.1186/1471-2105-10-74 (PMC2653488; doi:10.1186/1471-2105-10-74)
Supplement: Additional file 2 — RGG runner for Windows/Linux. Software to load and execute rgg files (Windows and Linux). [file 1471-2105-10-74-S2.zip › rggrunner/platform8/docs/swing-layout-1.0.3-doc.zip!/index-files/index-5.html]

G-Index


|  |  |  |
| --- | --- | --- |
| **Package** \| Class \| Use \| **Tree** \| **Deprecated** \| **Index** \| **Help** | |  |
| **PREV LETTER**   **NEXT LETTER** | **FRAMES**    **NO FRAMES**     **All Classes** |  |


A B C D G H I L M O P R S T U V 

---


## **G**

**getAutocreateContainerGaps()** - Method in class org.jdesktop.layout.GroupLayout: Returns whether or not gaps between the container and the first/last components should automatically be created. **getAutocreateContainerGaps()** - Method in class org.jdesktop.layout.GroupLayout: Returns whether or not gaps between the container and the first/last components should automatically be created. **getAutocreateGaps()** - Method in class org.jdesktop.layout.GroupLayout: Returns true if gaps between components are automatically be created. **getAutocreateGaps()** - Method in class org.jdesktop.layout.GroupLayout: Returns true if gaps between components are automatically be created. **getBaseline(JComponent)** - Static method in class org.jdesktop.layout.Baseline: Returns the baseline for the specified component, or -1 if the baseline can not be determined. **getBaseline(JComponent, int, int)** - Static method in class org.jdesktop.layout.Baseline: Returns the baseline for the specified component, or a value less than 0 if the baseline can not be determined. **getBaseline(JComponent)** - Static method in class org.jdesktop.layout.Baseline: Returns the baseline for the specified component, or -1 if the baseline can not be determined. **getBaseline(JComponent, int, int)** - Static method in class org.jdesktop.layout.Baseline: Returns the baseline for the specified component, or a value less than 0 if the baseline can not be determined. **getBaselineResizeBehavior(JComponent)** - Static method in class org.jdesktop.layout.Baseline: Returns a constant indicating how the baseline varies with the size of the component. **getBaselineResizeBehavior(JComponent)** - Static method in class org.jdesktop.layout.Baseline: Returns a constant indicating how the baseline varies with the size of the component. **getComponentBaseline(JComponent, int, int)** - Method in class org.jdesktop.layout.Baseline: Returns the baseline for the specified component, or -1 if the baseline can not be determined. **getComponentBaseline(JComponent, int, int)** - Method in class org.jdesktop.layout.Baseline: Returns the baseline for the specified component, or -1 if the baseline can not be determined. **getContainerGap(JComponent, int, Container)** - Method in class org.jdesktop.layout.LayoutStyle: Returns the amount of space to position a component inside its parent. **getContainerGap(JComponent, int, Container)** - Method in class org.jdesktop.layout.LayoutStyle: Returns the amount of space to position a component inside its parent. **getHonorsVisibility()** - Method in class org.jdesktop.layout.GroupLayout: Returns whether component visiblity is considered when sizing and positioning components. **getHonorsVisibility()** - Method in class org.jdesktop.layout.GroupLayout: Returns whether component visiblity is considered when sizing and positioning components. **getHorizontalGroup()** - Method in class org.jdesktop.layout.GroupLayout: Returns the `Group` that is responsible for layout along the horizontal axis. **getHorizontalGroup()** - Method in class org.jdesktop.layout.GroupLayout: Returns the `Group` that is responsible for layout along the horizontal axis. **getLayoutAlignmentX(Container)** - Method in class org.jdesktop.layout.GroupLayout: Returns the alignment along the x axis. **getLayoutAlignmentX(Container)** - Method in class org.jdesktop.layout.GroupLayout: Returns the alignment along the x axis. **getLayoutAlignmentY(Container)** - Method in class org.jdesktop.layout.GroupLayout: Returns the alignment along the y axis. **getLayoutAlignmentY(Container)** - Method in class org.jdesktop.layout.GroupLayout: Returns the alignment along the y axis. **getLayoutStyle()** - Method in class org.jdesktop.layout.GroupLayout: Returns the LayoutStyle instance to use **getLayoutStyle()** - Method in class org.jdesktop.layout.GroupLayout: Returns the LayoutStyle instance to use **getPreferredGap(JComponent, JComponent, int, int, Container)** - Method in class org.jdesktop.layout.LayoutStyle: Returns the amount of space to use between two components. **getPreferredGap(JComponent, JComponent, int, int, Container)** - Method in class org.jdesktop.layout.LayoutStyle: Returns the amount of space to use between two components. **getSharedInstance()** - Static method in class org.jdesktop.layout.LayoutStyle: Factory methods for obtaining the current `LayoutStyle` object appropriate for the current look and feel. **getSharedInstance()** - Static method in class org.jdesktop.layout.LayoutStyle: Factory methods for obtaining the current `LayoutStyle` object appropriate for the current look and feel. **getVerticalGroup()** - Method in class org.jdesktop.layout.GroupLayout: Returns the `ParallelGroup` that is responsible for layout along the vertical axis. **getVerticalGroup()** - Method in class org.jdesktop.layout.GroupLayout: Returns the `ParallelGroup` that is responsible for layout along the vertical axis. **GroupLayout** - Class in org.jdesktop.layout: GroupLayout is a LayoutManager that hierarchically groups components to achieve common, and not so common, layouts. **GroupLayout** - Class in org.jdesktop.layout: GroupLayout is a LayoutManager that hierarchically groups components to achieve common, and not so common, layouts. **GroupLayout(Container)** - Constructor for class org.jdesktop.layout.GroupLayout: Creates a GroupLayout for the specified JComponent. **GroupLayout(Container)** - Constructor for class org.jdesktop.layout.GroupLayout: Creates a GroupLayout for the specified JComponent. **GroupLayout.Group** - Class in org.jdesktop.layout: Group provides for commonality between the two types of operations supported by `GroupLayout`: laying out components one after another (`SequentialGroup`) or layout on top of each other (`ParallelGroup`). **GroupLayout.Group** - Class in org.jdesktop.layout: Group provides for commonality between the two types of operations supported by `GroupLayout`: laying out components one after another (`SequentialGroup`) or layout on top of each other (`ParallelGroup`). **GroupLayout.ParallelGroup** - Class in org.jdesktop.layout: A `Group` that lays out its elements on top of each other. **GroupLayout.ParallelGroup** - Class in org.jdesktop.layout: A `Group` that lays out its elements on top of each other. **GroupLayout.SequentialGroup** - Class in org.jdesktop.layout: A `Group` that lays out its elements sequentially, one after another. **GroupLayout.SequentialGroup** - Class in org.jdesktop.layout: A `Group` that lays out its elements sequentially, one after another.

---


|  |  |  |
| --- | --- | --- |
| **Package** \| Class \| Use \| **Tree** \| **Deprecated** \| **Index** \| **Help** | |  |
| **PREV LETTER**   **NEXT LETTER** | **FRAMES**    **NO FRAMES**     **All Classes** |  |


A B C D G H I L M O P R S T U V 

---
