# Supplementary material for: RGG: A general GUI Framework for R scripts
Source: BMC Bioinformatics. 2009 Mar 2;10:74. doi: 10.1186/1471-2105-10-74 (PMC2653488; doi:10.1186/1471-2105-10-74)
Supplement: Additional file 2 — RGG runner for Windows/Linux. Software to load and execute rgg files (Windows and Linux). [file 1471-2105-10-74-S2.zip › rggrunner/platform8/docs/swing-layout-1.0.3-doc.zip!/index-files/index-8.html]

L-Index


|  |  |  |
| --- | --- | --- |
| **Package** \| Class \| Use \| **Tree** \| **Deprecated** \| **Index** \| **Help** | |  |
| **PREV LETTER**   **NEXT LETTER** | **FRAMES**    **NO FRAMES**     **All Classes** |  |


A B C D G H I L M O P R S T U V 

---


## **L**

**layoutContainer(Container)** - Method in class org.jdesktop.layout.GroupLayout: Lays out the specified container. **layoutContainer(Container)** - Method in class org.jdesktop.layout.GroupLayout: Lays out the specified container. **LayoutStyle** - Class in org.jdesktop.layout: LayoutStyle is used to determine how much space to place between components during layout. **LayoutStyle** - Class in org.jdesktop.layout: LayoutStyle is used to determine how much space to place between components during layout. **LayoutStyle()** - Constructor for class org.jdesktop.layout.LayoutStyle: **LayoutStyle()** - Constructor for class org.jdesktop.layout.LayoutStyle: **LEADING** - Static variable in class org.jdesktop.layout.GroupLayout: Possible alignment type. **LEADING** - Static variable in class org.jdesktop.layout.GroupLayout: Possible alignment type. **linkSize(Component[])** - Method in class org.jdesktop.layout.GroupLayout: Forces the set of components to have the same size. **linkSize(Component[], int)** - Method in class org.jdesktop.layout.GroupLayout: Forces the set of components to have the same size. **linkSize(Component[])** - Method in class org.jdesktop.layout.GroupLayout: Forces the set of components to have the same size. **linkSize(Component[], int)** - Method in class org.jdesktop.layout.GroupLayout: Forces the set of components to have the same size.

---


|  |  |  |
| --- | --- | --- |
| **Package** \| Class \| Use \| **Tree** \| **Deprecated** \| **Index** \| **Help** | |  |
| **PREV LETTER**   **NEXT LETTER** | **FRAMES**    **NO FRAMES**     **All Classes** |  |


A B C D G H I L M O P R S T U V 

---
