# Supplementary material for: RGG: A general GUI Framework for R scripts
Source: BMC Bioinformatics. 2009 Mar 2;10:74. doi: 10.1186/1471-2105-10-74 (PMC2653488; doi:10.1186/1471-2105-10-74)
Supplement: Additional file 2 — RGG runner for Windows/Linux. Software to load and execute rgg files (Windows and Linux). [file 1471-2105-10-74-S2.zip › rggrunner/platform8/docs/swing-layout-1.0.3-doc.zip!/index.html]

Generated Documentation (Untitled)


<H2>
Frame Alert</H2>
<P>
This document is designed to be viewed using the frames feature. If you see this message, you are using a non-frame-capable web client.
<BR>
Link to<A HREF="org/jdesktop/layout/package-summary.html">Non-frame version.</A>
