# Supplementary material for: RGG: A general GUI Framework for R scripts
Source: BMC Bioinformatics. 2009 Mar 2;10:74. doi: 10.1186/1471-2105-10-74 (PMC2653488; doi:10.1186/1471-2105-10-74)
Supplement: Additional file 2 — RGG runner for Windows/Linux. Software to load and execute rgg files (Windows and Linux). [file 1471-2105-10-74-S2.zip › rggrunner/platform8/docs/swing-layout-1.0.3-doc.zip!/org/jdesktop/layout/Baseline.html]

Baseline


|  |  |  |
| --- | --- | --- |
| **Package** \| **Class** \| **Use** \| **Tree** \| **Deprecated** \| **Index** \| **Help** | |  |
| PREV CLASS   **NEXT CLASS** | **FRAMES**    **NO FRAMES**     **All Classes** |  |
| SUMMARY: NESTED \| FIELD \| CONSTR \| METHOD | DETAIL: FIELD \| CONSTR \| METHOD |  |


---


## org.jdesktop.layout Class Baseline

```
java.lang.Object
  org.jdesktop.layout.Baseline
```

---

``` public class Baseline extends java.lang.Object ```

Convenience class that can be used to determine the baseline of a
particular component. The static method `getBaseline` uses the
following algorithm to determine the baseline:

1. If the component has a `getBaseline(JComponent,int,int)`
   method, invoke it.- If there is a `UIManager` property of the name
     `Baseline.instance`, forward the call to that Baseline.- Otherwise use the built in support.

In addition to determining the baseline, this class also allows for
determining how the baseline changes as the size of the component changes.
The method getBaselineResizeBehavior can be used for this. This will return
one of BRB\_OTHER, BRB\_CONSTANT\_ASCENT, BRB\_CONSTANT\_DESCENT or
BRB\_CENTER\_OFFSET. The following algorithm is used in determining the
baseline resize behavior.

1. If the Component defines the method
   getBaselineResizeBehaviorInt, the return value from that method is used.- If running on 1.6, the Component method getBaselineResizeBehavior is
     invoked and the return value converted to one of the constants defined
     by this class.- If the component is one of the known Swing components,the baseline resize
       behavior is calculated and returned.- Otherwise, BRB\_OTHER is returned.

This class is primarily useful for JREs prior to 1.6. In 1.6 API for this
was added directly to Component. When run on 1.6 or newer, this class calls
into the appropriate Component methods.

---

| **Field Summary** | |
| --- | --- |
| `static int` | `BRB_CENTER_OFFSET`             Baseline resize behavior constant. |
| `static int` | `BRB_CONSTANT_ASCENT`             Baseline resize behavior constant. |
| `static int` | `BRB_CONSTANT_DESCENT`             Baseline resize behavior constant. |
| `static int` | `BRB_OTHER`             Baseline resize behavior constant. |


| **Constructor Summary** | |
| --- | --- |
| `protected` | `Baseline()`             Creates an instance of Baseline. |


| **Method Summary** | |
| --- | --- |
| `static int` | `getBaseline(javax.swing.JComponent component)`             Returns the baseline for the specified component, or -1 if the baseline can not be determined. |
| `static int` | `getBaseline(javax.swing.JComponent component, int width, int height)`             Returns the baseline for the specified component, or a value less than 0 if the baseline can not be determined. |
| `static int` | `getBaselineResizeBehavior(javax.swing.JComponent c)`             Returns a constant indicating how the baseline varies with the size of the component. |
| `int` | `getComponentBaseline(javax.swing.JComponent component, int width, int height)`             Returns the baseline for the specified component, or -1 if the baseline can not be determined. |

**Methods inherited from class java.lang.Object**

`clone, equals, finalize, getClass, hashCode, notify, notifyAll, toString, wait, wait, wait`

**Field Detail**

### BRB\_CONSTANT\_ASCENT

```
public static final int BRB_CONSTANT_ASCENT
```

:   Baseline resize behavior constant. Indicates as the size of the component
    changes the baseline remains a fixed distance from the top of the
    component.

    **See Also:**: Constant Field Values

---


### BRB\_CONSTANT\_DESCENT

```
public static final int BRB_CONSTANT_DESCENT
```

:   Baseline resize behavior constant. Indicates as the size of the component
    changes the baseline remains a fixed distance from the bottom of the
    component.

    **See Also:**: Constant Field Values

---


### BRB\_CENTER\_OFFSET

```
public static final int BRB_CENTER_OFFSET
```

:   Baseline resize behavior constant. Indicates as the size of the component
    changes the baseline remains a fixed distance from the center of the
    component.

    **See Also:**: Constant Field Values

---


### BRB\_OTHER

```
public static final int BRB_OTHER
```

:   Baseline resize behavior constant. Indicates as the size of the component
    changes the baseline can not be determined using one of the other
    constants.

    **See Also:**: Constant Field Values


**Constructor Detail**

### Baseline

```
protected Baseline()
```

:   Creates an instance of Baseline. You typically don't create a
    Baseline. The constructor is provided by look and feels that wish
    to provide baseline support.

    A custom look and feel that wants to provide `Baseline`
    support should put the instance in the defaults returned
    from `getDefaults`. If you want to override the
    baseline suport for a look and feel place the instance in the defaults
    returned from UIManager.getLookAndFeelDefaults(). Tthis will ensure
    that if the look and feel changes the appropriate baseline can be used.


**Method Detail**

### getBaselineResizeBehavior

```
public static int getBaselineResizeBehavior(javax.swing.JComponent c)
```

:   Returns a constant indicating how the baseline varies with the size
    of the component.

    :   **Parameters:**: `c` - the JComponent to get the baseline resize behavior for **Returns:**: one of BRB\_CONSTANT\_ASCENT, BRB\_CONSTANT\_DESCENT, BRB\_CENTER\_OFFSET or BRB\_OTHER

---


### getBaseline

```
public static int getBaseline(javax.swing.JComponent component)
```

:   Returns the baseline for the specified component, or -1 if the
    baseline can not be determined. The baseline is measured from
    the top of the component. This method returns the baseline based
    on the preferred size.

    :   **Parameters:**: `component` - JComponent to calculate baseline for **Returns:**: baseline for the specified component

---


### getBaseline

```
public static int getBaseline(javax.swing.JComponent component,
                              int width,
                              int height)
```

:   Returns the baseline for the specified component, or a value less
    than 0 if the baseline can not be determined. The baseline is measured
    from the top of the component.

    :   **Parameters:**: `component` - JComponent to calculate baseline for: `width` - Width of the component to determine baseline for.: `height` - Height of the component to determine baseline for. **Returns:**: baseline for the specified component

---


### getComponentBaseline

```
public int getComponentBaseline(javax.swing.JComponent component,
                                int width,
                                int height)
```

:   Returns the baseline for the specified component, or -1 if the
    baseline can not be determined. The baseline is measured from
    the top of the component.

    :   **Parameters:**: `component` - JComponent to calculate baseline for: `width` - Width of the component to determine baseline for.: `height` - Height of the component to determine baseline for. **Returns:**: baseline for the specified component


---


|  |  |  |
| --- | --- | --- |
| **Package** \| **Class** \| **Use** \| **Tree** \| **Deprecated** \| **Index** \| **Help** | |  |
| PREV CLASS   **NEXT CLASS** | **FRAMES**    **NO FRAMES**     **All Classes** |  |
| SUMMARY: NESTED \| FIELD \| CONSTR \| METHOD | DETAIL: FIELD \| CONSTR \| METHOD |  |


---
