# Supplementary material for: RGG: A general GUI Framework for R scripts
Source: BMC Bioinformatics. 2009 Mar 2;10:74. doi: 10.1186/1471-2105-10-74 (PMC2653488; doi:10.1186/1471-2105-10-74)
Supplement: Additional file 2 — RGG runner for Windows/Linux. Software to load and execute rgg files (Windows and Linux). [file 1471-2105-10-74-S2.zip › rggrunner/platform8/docs/swing-layout-1.0.3-doc.zip!/org/jdesktop/layout/GroupLayout.Group.html]

GroupLayout.Group


|  |  |  |
| --- | --- | --- |
| **Package** \| **Class** \| **Use** \| **Tree** \| **Deprecated** \| **Index** \| **Help** | |  |
| **PREV CLASS**   **NEXT CLASS** | **FRAMES**    **NO FRAMES**     **All Classes** |  |
| SUMMARY: NESTED \| FIELD \| CONSTR \| METHOD | DETAIL: FIELD \| CONSTR \| METHOD |  |


---


## org.jdesktop.layout Class GroupLayout.Group

```
java.lang.Object
  org.jdesktop.layout.GroupLayout.Group
```

**Direct Known Subclasses:**: GroupLayout.ParallelGroup, GroupLayout.SequentialGroup

**Enclosing class:**: GroupLayout

---

``` public abstract class GroupLayout.Group extends java.lang.Object ```

Group provides for commonality between the two types of operations
supported by `GroupLayout`: laying out components one
after another (`SequentialGroup`) or layout on top
of each other (`ParallelGroup`). Use one of
`createSequentialGroup` or
`createParallelGroup` to create one.

---

**Method Summary**

**Methods inherited from class java.lang.Object**

`clone, equals, finalize, getClass, hashCode, notify, notifyAll, toString, wait, wait, wait`

---


|  |  |  |
| --- | --- | --- |
| **Package** \| **Class** \| **Use** \| **Tree** \| **Deprecated** \| **Index** \| **Help** | |  |
| **PREV CLASS**   **NEXT CLASS** | **FRAMES**    **NO FRAMES**     **All Classes** |  |
| SUMMARY: NESTED \| FIELD \| CONSTR \| METHOD | DETAIL: FIELD \| CONSTR \| METHOD |  |


---
