# Supplementary material for: RGG: A general GUI Framework for R scripts
Source: BMC Bioinformatics. 2009 Mar 2;10:74. doi: 10.1186/1471-2105-10-74 (PMC2653488; doi:10.1186/1471-2105-10-74)
Supplement: Additional file 2 — RGG runner for Windows/Linux. Software to load and execute rgg files (Windows and Linux). [file 1471-2105-10-74-S2.zip › rggrunner/platform8/docs/swing-layout-1.0.3-doc.zip!/org/jdesktop/layout/GroupLayout.ParallelGroup.html]

GroupLayout.ParallelGroup


|  |  |  |
| --- | --- | --- |
| **Package** \| **Class** \| **Use** \| **Tree** \| **Deprecated** \| **Index** \| **Help** | |  |
| **PREV CLASS**   **NEXT CLASS** | **FRAMES**    **NO FRAMES**     **All Classes** |  |
| SUMMARY: NESTED \| FIELD \| CONSTR \| METHOD | DETAIL: FIELD \| CONSTR \| METHOD |  |


---


## org.jdesktop.layout Class GroupLayout.ParallelGroup

```
java.lang.Object
  org.jdesktop.layout.GroupLayout.Group
      org.jdesktop.layout.GroupLayout.ParallelGroup
```

**Enclosing class:**: GroupLayout

---

``` public class GroupLayout.ParallelGroup extends GroupLayout.Group ```

A `Group` that lays out its elements on top of each
other. If a child element is smaller than the provided space it
is aligned based on the alignment of the child (if specified) or
on the alignment of the ParallelGroup.

**See Also:**: `GroupLayout.createParallelGroup()`

---

| **Method Summary** | |
| --- | --- |
| `GroupLayout.ParallelGroup` | `add(java.awt.Component component)`             Adds the specified Component. |
| `GroupLayout.ParallelGroup` | `add(java.awt.Component component, int min, int pref, int max)`             Adds the specified `Component`. |
| `GroupLayout.ParallelGroup` | `add(GroupLayout.Group group)`             Adds the specified `Group`. |
| `GroupLayout.ParallelGroup` | `add(int pref)`             Adds a rigid gap. |
| `GroupLayout.ParallelGroup` | `add(int alignment, java.awt.Component component)`             Adds the specified Component. |
| `GroupLayout.ParallelGroup` | `add(int alignment, java.awt.Component component, int min, int pref, int max)`             Adds the specified `Component`. |
| `GroupLayout.ParallelGroup` | `add(int alignment, GroupLayout.Group group)`             Adds the specified `Group` as a child of this group. |
| `GroupLayout.ParallelGroup` | `add(int min, int pref, int max)`             Adds a gap with the specified size. |

**Methods inherited from class java.lang.Object**

`clone, equals, finalize, getClass, hashCode, notify, notifyAll, toString, wait, wait, wait`

**Method Detail**

### add

```
public GroupLayout.ParallelGroup add(GroupLayout.Group group)
```

:   Adds the specified `Group`.

    :   **Parameters:**: `group` - the Group to add **Returns:**: this Group

---


### add

```
public GroupLayout.ParallelGroup add(java.awt.Component component)
```

:   Adds the specified Component. If the Component's min/max
    are different from its pref than the component will be resizable.

    :   **Parameters:**: `component` - the Component to add **Returns:**: this `ParallelGroup`

---


### add

```
public GroupLayout.ParallelGroup add(java.awt.Component component,
                                     int min,
                                     int pref,
                                     int max)
```

:   Adds the specified `Component`. Min, pref and max
    can be absolute values, or they can be one of
    `DEFAULT_SIZE` or `PREFERRED_SIZE`. For
    example, the following:

    ```
       add(component, PREFERRED_SIZE, PREFERRED_SIZE, 1000);
    ```

    Forces a max of 1000, with the min and preferred equalling that
    of the preferred size of `component`.

    :   **Parameters:**: `component` - the Component to add: `min` - the minimum size: `pref` - the preferred size: `max` - the maximum size **Returns:**: this `SequentialGroup` **Throws:**: `java.lang.IllegalArgumentException` - if min, pref or max are not positive and not one of PREFERRED\_SIZE or DEFAULT\_SIZE.

---


### add

```
public GroupLayout.ParallelGroup add(int pref)
```

:   Adds a rigid gap.

    :   **Parameters:**: `pref` - the size of the gap **Returns:**: this `ParallelGroup` **Throws:**: `java.lang.IllegalArgumentException` - if min < 0 or pref < 0 or max < 0 or the following is not meant min <= pref <= max.

---


### add

```
public GroupLayout.ParallelGroup add(int min,
                                     int pref,
                                     int max)
```

:   Adds a gap with the specified size.

    :   **Parameters:**: `min` - the minimum size of the gap: `pref` - the preferred size of the gap: `max` - the maximum size of the gap **Returns:**: this `ParallelGroup` **Throws:**: `java.lang.IllegalArgumentException` - if min < 0 or pref < 0 or max < 0 or the following is not meant min <= pref <= max.

---


### add

```
public GroupLayout.ParallelGroup add(int alignment,
                                     GroupLayout.Group group)
```

:   Adds the specified `Group` as a child of this group.

    :   **Parameters:**: `alignment` - the alignment of the Group.: `group` - the Group to add **Returns:**: this `ParallelGroup` **Throws:**: `java.lang.IllegalArgumentException` - if alignment is not one of `LEADING`, `TRAILING` or `CENTER`

---


### add

```
public GroupLayout.ParallelGroup add(int alignment,
                                     java.awt.Component component)
```

:   Adds the specified Component. If the Component's min/max
    are different from its pref than the component will be resizable.

    :   **Parameters:**: `alignment` - the alignment for the component: `component` - the Component to add **Returns:**: this `Group` **Throws:**: `java.lang.IllegalArgumentException` - if alignment is not one of `LEADING`, `TRAILING` or `CENTER`

---


### add

```
public GroupLayout.ParallelGroup add(int alignment,
                                     java.awt.Component component,
                                     int min,
                                     int pref,
                                     int max)
```

:   Adds the specified `Component`. Min, pref and max
    can be absolute values, or they can be one of
    `DEFAULT_SIZE` or `PREFERRED_SIZE`. For
    example, the following:

    ```
       add(component, PREFERRED_SIZE, PREFERRED_SIZE, 1000);
    ```

    Forces a max of 1000, with the min and preferred equalling that
    of the preferred size of `component`.

    :   **Parameters:**: `alignment` - the alignment for the component.: `component` - the Component to add: `min` - the minimum size: `pref` - the preferred size: `max` - the maximum size **Returns:**: this `Group` **Throws:**: `java.lang.IllegalArgumentException` - if min, pref or max are not positive and not one of PREFERRED\_SIZE or DEFAULT\_SIZE.


---


|  |  |  |
| --- | --- | --- |
| **Package** \| **Class** \| **Use** \| **Tree** \| **Deprecated** \| **Index** \| **Help** | |  |
| **PREV CLASS**   **NEXT CLASS** | **FRAMES**    **NO FRAMES**     **All Classes** |  |
| SUMMARY: NESTED \| FIELD \| CONSTR \| METHOD | DETAIL: FIELD \| CONSTR \| METHOD |  |


---
