# Supplementary material for: RGG: A general GUI Framework for R scripts
Source: BMC Bioinformatics. 2009 Mar 2;10:74. doi: 10.1186/1471-2105-10-74 (PMC2653488; doi:10.1186/1471-2105-10-74)
Supplement: Additional file 2 — RGG runner for Windows/Linux. Software to load and execute rgg files (Windows and Linux). [file 1471-2105-10-74-S2.zip › rggrunner/platform8/docs/swing-layout-1.0.3-doc.zip!/org/jdesktop/layout/GroupLayout.SequentialGroup.html]

GroupLayout.SequentialGroup


|  |  |  |
| --- | --- | --- |
| **Package** \| **Class** \| **Use** \| **Tree** \| **Deprecated** \| **Index** \| **Help** | |  |
| **PREV CLASS**   **NEXT CLASS** | **FRAMES**    **NO FRAMES**     **All Classes** |  |
| SUMMARY: NESTED \| FIELD \| CONSTR \| METHOD | DETAIL: FIELD \| CONSTR \| METHOD |  |


---


## org.jdesktop.layout Class GroupLayout.SequentialGroup

```
java.lang.Object
  org.jdesktop.layout.GroupLayout.Group
      org.jdesktop.layout.GroupLayout.SequentialGroup
```

**Enclosing class:**: GroupLayout

---

``` public class GroupLayout.SequentialGroup extends GroupLayout.Group ```

A `Group` that lays out its elements sequentially, one
after another. This class has no public constructor, use the
`createSequentialGroup` method to create one.

**See Also:**: `GroupLayout.createSequentialGroup()`

---

| **Method Summary** | |
| --- | --- |
| `GroupLayout.SequentialGroup` | `add(boolean useAsBaseline, java.awt.Component component)`             Adds a `Component` to this `Group`. |
| `GroupLayout.SequentialGroup` | `add(boolean useAsBaseline, java.awt.Component component, int min, int pref, int max)`             Adds a `Component` to this `Group` with the specified size. |
| `GroupLayout.SequentialGroup` | `add(boolean useAsBaseline, GroupLayout.Group group)`             Adds a `Group` to this `Group`. |
| `GroupLayout.SequentialGroup` | `add(java.awt.Component component)`             Adds the specified Component. |
| `GroupLayout.SequentialGroup` | `add(java.awt.Component component, int min, int pref, int max)`             Adds the specified `Component`. |
| `GroupLayout.SequentialGroup` | `add(GroupLayout.Group group)`             Adds the specified `Group` to this `SequentialGroup` |
| `GroupLayout.SequentialGroup` | `add(int pref)`             Adds a rigid gap. |
| `GroupLayout.SequentialGroup` | `add(int min, int pref, int max)`             Adds a gap with the specified size. |
| `GroupLayout.SequentialGroup` | `addContainerGap()`             Adds an element representing the preferred gap between one edge of the container and the next/previous Component. |
| `GroupLayout.SequentialGroup` | `addContainerGap(int pref, int max)`             Adds an element representing the preferred gap between one edge of the container and the next/previous Component. |
| `GroupLayout.SequentialGroup` | `addPreferredGap(int type)`             Adds an element representing the preferred gap between the nearest components. |
| `GroupLayout.SequentialGroup` | `addPreferredGap(int type, int pref, int max)`             Adds an element for the preferred gap between the nearest components. |
| `GroupLayout.SequentialGroup` | `addPreferredGap(javax.swing.JComponent comp1, javax.swing.JComponent comp2, int type)`             Adds an element representing the preferred gap between the two components. |
| `GroupLayout.SequentialGroup` | `addPreferredGap(javax.swing.JComponent comp1, javax.swing.JComponent comp2, int type, boolean canGrow)`             Adds an element representing the preferred gap between the two components. |

**Methods inherited from class java.lang.Object**

`clone, equals, finalize, getClass, hashCode, notify, notifyAll, toString, wait, wait, wait`

**Method Detail**

### add

```
public GroupLayout.SequentialGroup add(GroupLayout.Group group)
```

:   Adds the specified `Group` to this
    `SequentialGroup`

    :   **Parameters:**: `group` - the Group to add **Returns:**: this Group

---


### add

```
public GroupLayout.SequentialGroup add(boolean useAsBaseline,
                                       GroupLayout.Group group)
```

:   Adds a `Group` to this `Group`.

    :   **Parameters:**: `group` - the `Group` to add: `useAsBaseline` - whether the specified `Group` should be used to calculate the baseline for this `Group` **Returns:**: this `Group`

---


### add

```
public GroupLayout.SequentialGroup add(java.awt.Component component)
```

:   Adds the specified Component. If the Component's min/max
    are different from its pref than the component will be resizable.

    :   **Parameters:**: `component` - the Component to add **Returns:**: this `SequentialGroup`

---


### add

```
public GroupLayout.SequentialGroup add(boolean useAsBaseline,
                                       java.awt.Component component)
```

:   Adds a `Component` to this `Group`.

    :   **Parameters:**: `useAsBaseline` - whether the specified `Component` should be used to calculate the baseline for this `Group`: `component` - the `Component` to add **Returns:**: this `Group`

---


### add

```
public GroupLayout.SequentialGroup add(java.awt.Component component,
                                       int min,
                                       int pref,
                                       int max)
```

:   Adds the specified `Component`. Min, pref and max
    can be absolute values, or they can be one of
    `DEFAULT_SIZE` or `PREFERRED_SIZE`. For
    example, the following:

    ```
       add(component, PREFERRED_SIZE, PREFERRED_SIZE, 1000);
    ```

    Forces a max of 1000, with the min and preferred equalling that
    of the preferred size of `component`.

    :   **Parameters:**: `component` - the Component to add: `min` - the minimum size: `pref` - the preferred size: `max` - the maximum size **Returns:**: this `SequentialGroup` **Throws:**: `java.lang.IllegalArgumentException` - if min, pref or max are not positive and not one of PREFERRED\_SIZE or DEFAULT\_SIZE

---


### add

```
public GroupLayout.SequentialGroup add(boolean useAsBaseline,
                                       java.awt.Component component,
                                       int min,
                                       int pref,
                                       int max)
```

:   Adds a `Component` to this `Group`
    with the specified size.

    :   **Parameters:**: `useAsBaseline` - whether the specified `Component` should be used to calculate the baseline for this `Group`: `component` - the `Component` to add: `min` - the minimum size or one of `DEFAULT_SIZE` or `PREFERRED_SIZE`: `pref` - the preferred size or one of `DEFAULT_SIZE` or `PREFERRED_SIZE`: `max` - the maximum size or one of `DEFAULT_SIZE` or `PREFERRED_SIZE` **Returns:**: this `Group`

---


### add

```
public GroupLayout.SequentialGroup add(int pref)
```

:   Adds a rigid gap.

    :   **Parameters:**: `pref` - the size of the gap **Returns:**: this `SequentialGroup` **Throws:**: `java.lang.IllegalArgumentException` - if min < 0 or pref < 0 or max < 0 or the following is not meant min <= pref <= max

---


### add

```
public GroupLayout.SequentialGroup add(int min,
                                       int pref,
                                       int max)
```

:   Adds a gap with the specified size.

    :   **Parameters:**: `min` - the minimum size of the gap, or PREFERRED\_SIZE: `pref` - the preferred size of the gap: `max` - the maximum size of the gap, or PREFERRED\_SIZE **Returns:**: this `SequentialGroup` **Throws:**: `java.lang.IllegalArgumentException` - if min < 0 or pref < 0 or max < 0 or the following is not meant min <= pref <= max

---


### addPreferredGap

```
public GroupLayout.SequentialGroup addPreferredGap(javax.swing.JComponent comp1,
                                                   javax.swing.JComponent comp2,
                                                   int type)
```

:   Adds an element representing the preferred gap between the two
    components.

    :   **Parameters:**: `comp1` - the first component: `comp2` - the second component: `type` - the type of gap; one of the constants defined by LayoutStyle **Returns:**: this `SequentialGroup` **Throws:**: `java.lang.IllegalArgumentException` - if `type` is not a valid LayoutStyle constant **See Also:**: `LayoutStyle`

---


### addPreferredGap

```
public GroupLayout.SequentialGroup addPreferredGap(javax.swing.JComponent comp1,
                                                   javax.swing.JComponent comp2,
                                                   int type,
                                                   boolean canGrow)
```

:   Adds an element representing the preferred gap between the two
    components.

    :   **Parameters:**: `comp1` - the first component: `comp2` - the second component: `type` - the type of gap; one of the constants defined by LayoutStyle: `canGrow` - true if the gap can grow if more space is available **Returns:**: this `SequentialGroup` **Throws:**: `java.lang.IllegalArgumentException` - if `type` is not a valid LayoutStyle constant **See Also:**: `LayoutStyle`

---


### addPreferredGap

```
public GroupLayout.SequentialGroup addPreferredGap(int type)
```

:   Adds an element representing the preferred gap between the
    nearest components. That is, during layout the neighboring
    components are found, and the min, pref and max of this
    element is set based on the preferred gap between the
    components. If no neighboring components are found the
    min, pref and max are set to 0.

    :   **Parameters:**: `type` - the type of gap; one of the LayoutStyle constants **Returns:**: this SequentialGroup **Throws:**: `java.lang.IllegalArgumentException` - if type is not one of `LayoutStyle.RELATED` or `LayoutStyle.UNRELATED` **See Also:**: `LayoutStyle`

---


### addPreferredGap

```
public GroupLayout.SequentialGroup addPreferredGap(int type,
                                                   int pref,
                                                   int max)
```

:   Adds an element for the preferred gap between the
    nearest components. That is, during layout the neighboring
    components are found, and the min of this
    element is set based on the preferred gap between the
    components. If no neighboring components are found the
    min is set to 0. This method allows you to specify the
    preferred and maximum size by way of the `pref`
    and `max` arguments. These can either be a
    value >= 0, in which case the preferred or max is the max
    of the argument and the preferred gap, of DEFAULT\_VALUE in
    which case the value is the same as the preferred gap.

    :   **Parameters:**: `type` - the type of gap; one of LayoutStyle.RELATED or LayoutStyle.UNRELATED: `pref` - the preferred size; one of DEFAULT\_SIZE or a value > 0: `max` - the maximum size; one of DEFAULT\_SIZE, PREFERRED\_SIZE or a value > 0 **Returns:**: this SequentialGroup **Throws:**: `java.lang.IllegalArgumentException` - if type is not one of `LayoutStyle.RELATED` or `LayoutStyle.UNRELATED` or pref/max is != DEFAULT\_SIZE and < 0, or pref > max **See Also:**: `LayoutStyle`

---


### addContainerGap

```
public GroupLayout.SequentialGroup addContainerGap()
```

:   Adds an element representing the preferred gap between one edge
    of the container and the next/previous Component. This will have
    no effect if the next/previous element is not a Component and does
    not touch one edge of the parent container.

    :   **Returns:**: this `SequentialGroup`.

---


### addContainerGap

```
public GroupLayout.SequentialGroup addContainerGap(int pref,
                                                   int max)
```

:   Adds an element representing the preferred gap between one edge
    of the container and the next/previous Component. This will have
    no effect if the next/previous element is not a Component and does
    not touch one edge of the parent container.

    :   **Parameters:**: `pref` - the preferred size; one of DEFAULT\_SIZE or a value > 0: `max` - the maximum size; one of DEFAULT\_SIZE, PREFERRED\_SIZE or a value > 0. **Returns:**: this `SequentialGroup` **Throws:**: `java.lang.IllegalArgumentException` - if pref/max is != DEFAULT\_SIZE and < 0, or pref > max


---


|  |  |  |
| --- | --- | --- |
| **Package** \| **Class** \| **Use** \| **Tree** \| **Deprecated** \| **Index** \| **Help** | |  |
| **PREV CLASS**   **NEXT CLASS** | **FRAMES**    **NO FRAMES**     **All Classes** |  |
| SUMMARY: NESTED \| FIELD \| CONSTR \| METHOD | DETAIL: FIELD \| CONSTR \| METHOD |  |


---
