# Supplementary material for: RGG: A general GUI Framework for R scripts
Source: BMC Bioinformatics. 2009 Mar 2;10:74. doi: 10.1186/1471-2105-10-74 (PMC2653488; doi:10.1186/1471-2105-10-74)
Supplement: Additional file 2 — RGG runner for Windows/Linux. Software to load and execute rgg files (Windows and Linux). [file 1471-2105-10-74-S2.zip › rggrunner/platform8/docs/swing-layout-1.0.3-doc.zip!/org/jdesktop/layout/GroupLayout.html]

GroupLayout


|  |  |  |
| --- | --- | --- |
| **Package** \| **Class** \| **Use** \| **Tree** \| **Deprecated** \| **Index** \| **Help** | |  |
| **PREV CLASS**   **NEXT CLASS** | **FRAMES**    **NO FRAMES**     **All Classes** |  |
| SUMMARY: NESTED \| FIELD \| CONSTR \| METHOD | DETAIL: FIELD \| CONSTR \| METHOD |  |


---


## org.jdesktop.layout Class GroupLayout

```
java.lang.Object
  org.jdesktop.layout.GroupLayout
```

**All Implemented Interfaces:**: java.awt.LayoutManager, java.awt.LayoutManager2

---

``` public class GroupLayout extends java.lang.Object implements java.awt.LayoutManager2 ```

GroupLayout is a LayoutManager that hierarchically groups components to
achieve common, and not so common, layouts. Grouping is done by instances
of the Group class. GroupLayout supports two types of groups:

|  |  |  |  |
| --- | --- | --- | --- |
| Sequential: A sequential group positions its child elements sequentially, one after another.|  |  | | --- | --- | | Parallel: A parallel group positions its child elements in the same space on top of each other. Parallel groups can also align the child elements along their baseline. | | | |

Each Group can contain any number of child groups, Components or gaps.
GroupLayout treats each axis independently. That is, there is a group
representing the horizontal axis, and a separate group representing the
vertical axis. The horizontal group is responsible for setting the x
and width of its contents, where as the vertical group is responsible for
setting the y and height of its contents.

The following code builds a simple layout consisting of two labels in
one column, followed by two textfields in the next column:

```
   JComponent panel = ...;
   GroupLayout layout = new GroupLayout(panel);
   panel.setLayout(layout);
   layout.setAutocreateGaps(true);
   layout.setAutocreateContainerGaps(true);
   GroupLayout.SequentialGroup hGroup = layout.createSequentialGroup();
   hGroup.add(layout.createParallelGroup().add(label1).add(label2)).
          add(layout.createParallelGroup().add(tf1).add(tf2));
   layout.setHorizontalGroup(hGroup);
   GroupLayout.SequentialGroup vGroup = layout.createSequentialGroup();
   vGroup.add(layout.createParallelGroup(GroupLayout.BASELINE).add(label1).add(tf1)).
          add(layout.createParallelGroup(GroupLayout.BASELINE).add(label2).add(tf2));
   layout.setVerticalGroup(vGroup);
```

This layout consists of the following:

- The horizontal axis consists of a sequential group containing two
  parallel groups. The first parallel group consists of the labels,
  with the second parallel group consisting of the text fields.- The vertical axis similarly consists of a sequential group
    containing two parallel groups. The parallel groups align their
    contents along the baseline. The first parallel group consists
    of the first label and text field, and the second group consists
    of the second label and text field.

There are a couple of things to notice in this code:

- You need not explicitly add the components to the container, this
  is indirectly done by using one of the `add` methods.- The various `add` methods of `Groups` return
    themselves. This allows for easy chaining of invocations. For
    example, `group.add(label1).add(label2);` is equivalent to
    `group.add(label1);group.add(label2);`.- There are no public constructors for the Groups, instead
      use the create methods of `GroupLayout`.

GroupLayout offer the ability to automatically insert the appropriate gap
between components. This can be turned on using the
`setAutocreateGaps()` method. Similarly you can use
the `setAutocreateContainerGaps()` method to insert gaps
between the components and the container.

---

| **Nested Class Summary** | |
| --- | --- |
| `class` | `GroupLayout.Group`             Group provides for commonality between the two types of operations supported by `GroupLayout`: laying out components one after another (`SequentialGroup`) or layout on top of each other (`ParallelGroup`). |
| `class` | `GroupLayout.ParallelGroup`             A `Group` that lays out its elements on top of each other. |
| `class` | `GroupLayout.SequentialGroup`             A `Group` that lays out its elements sequentially, one after another. |


| **Field Summary** | |
| --- | --- |
| `static int` | `BASELINE`             Possible alignment type. |
| `static int` | `CENTER`             Possible alignment type. |
| `static int` | `DEFAULT_SIZE`             Possible value for the add methods that takes a Component. |
| `static int` | `HORIZONTAL`             Possible argument when linking sizes of components. |
| `static int` | `LEADING`             Possible alignment type. |
| `static int` | `PREFERRED_SIZE`             Possible value for the add methods that takes a Component. |
| `static int` | `TRAILING`             Possible alignment type. |
| `static int` | `VERTICAL`             Possible argument when linking sizes of components. |


**Constructor Summary**

`GroupLayout(java.awt.Container host)`
  
          Creates a GroupLayout for the specified JComponent.


| **Method Summary** | |
| --- | --- |
| `void` | `addLayoutComponent(java.awt.Component component, java.lang.Object constraints)`             Notification that a `Component` has been added to the parent container. |
| `void` | `addLayoutComponent(java.lang.String name, java.awt.Component component)`             Notification that a `Component` has been added to the parent container. |
| `GroupLayout.ParallelGroup` | `createBaselineGroup(boolean resizable, boolean anchorBaselineToTop)`             Creates and returns a `ParallelGroup` that aligns it's elements along the baseline. |
| `GroupLayout.ParallelGroup` | `createParallelGroup()`             Creates and returns a `ParallelGroup` with a `LEADING` alignment. |
| `GroupLayout.ParallelGroup` | `createParallelGroup(int alignment)`             Creates and returns an `ParallelGroup`. |
| `GroupLayout.ParallelGroup` | `createParallelGroup(int alignment, boolean resizable)`             Creates and returns an `ParallelGroup`. |
| `GroupLayout.SequentialGroup` | `createSequentialGroup()`             Creates and returns a `SequentialGroup`. |
| `boolean` | `getAutocreateContainerGaps()`             Returns whether or not gaps between the container and the first/last components should automatically be created. |
| `boolean` | `getAutocreateGaps()`             Returns true if gaps between components are automatically be created. |
| `boolean` | `getHonorsVisibility()`             Returns whether component visiblity is considered when sizing and positioning components. |
| `GroupLayout.Group` | `getHorizontalGroup()`             Returns the `Group` that is responsible for layout along the horizontal axis. |
| `float` | `getLayoutAlignmentX(java.awt.Container parent)`             Returns the alignment along the x axis. |
| `float` | `getLayoutAlignmentY(java.awt.Container parent)`             Returns the alignment along the y axis. |
| `LayoutStyle` | `getLayoutStyle()`             Returns the LayoutStyle instance to use |
| `GroupLayout.Group` | `getVerticalGroup()`             Returns the `ParallelGroup` that is responsible for layout along the vertical axis. |
| `void` | `invalidateLayout(java.awt.Container parent)`             Invalidates the layout, indicating that if the layout manager has cached information it should be discarded. |
| `void` | `layoutContainer(java.awt.Container parent)`             Lays out the specified container. |
| `void` | `linkSize(java.awt.Component[] components)`             Forces the set of components to have the same size. |
| `void` | `linkSize(java.awt.Component[] components, int axis)`             Forces the set of components to have the same size. |
| `java.awt.Dimension` | `maximumLayoutSize(java.awt.Container parent)`             Returns the maximum size for the specified container. |
| `java.awt.Dimension` | `minimumLayoutSize(java.awt.Container parent)`             Returns the minimum size for the specified container. |
| `java.awt.Dimension` | `preferredLayoutSize(java.awt.Container parent)`             Returns the preferred size for the specified container. |
| `void` | `removeLayoutComponent(java.awt.Component component)`             Notification that a `Component` has been removed from the parent container. |
| `void` | `replace(java.awt.Component existingComponent, java.awt.Component newComponent)`             Removes an existing component replacing it with the specified component. |
| `void` | `setAutocreateContainerGaps(boolean autocreatePadding)`             Sets whether or not gaps between the container and the first/last components should automatically be created. |
| `void` | `setAutocreateGaps(boolean autocreatePadding)`             Sets whether or not a gap between components should automatically be created. |
| `void` | `setHonorsVisibility(boolean honorsVisibility)`             Sets whether component visiblity is considered when sizing and positioning components. |
| `void` | `setHonorsVisibility(java.awt.Component component, java.lang.Boolean honorsVisibility)`             Sets whether the component's visiblity is considered for sizing and positioning. |
| `void` | `setHorizontalGroup(GroupLayout.Group group)`             Sets the `Group` that is responsible for layout along the horizontal axis. |
| `void` | `setLayoutStyle(LayoutStyle layoutStyle)`             Sets the LayoutStyle this GroupLayout is to use. |
| `void` | `setVerticalGroup(GroupLayout.Group group)`             Sets the `Group` that is responsible for layout along the vertical axis. |
| `java.lang.String` | `toString()`             Returns a textual description of this GroupLayout. |

**Methods inherited from class java.lang.Object**

`clone, equals, finalize, getClass, hashCode, notify, notifyAll, wait, wait, wait`

**Field Detail**

### HORIZONTAL

```
public static final int HORIZONTAL
```

:   Possible argument when linking sizes of components. Specifies the
    the two component should share the same size along the horizontal
    axis.

    **See Also:**: `linkSize(java.awt.Component[],int)`, Constant Field Values

---


### VERTICAL

```
public static final int VERTICAL
```

:   Possible argument when linking sizes of components. Specifies the
    the two component should share the same size along the vertical
    axis.

    **See Also:**: `linkSize(java.awt.Component[],int)`, Constant Field Values

---


### LEADING

```
public static final int LEADING
```

:   Possible alignment type. Indicates the elements should be
    aligned to the origin. For the horizontal axis with a left to
    right orientation this means aligned to the left.

    **See Also:**: `createParallelGroup(int)`, Constant Field Values

---


### TRAILING

```
public static final int TRAILING
```

:   Possible alignment type. Indicates the elements should be
    aligned to the end. For the horizontal axis with a left to
    right orientation this means aligned to the right.

    **See Also:**: `createParallelGroup(int)`, Constant Field Values

---


### CENTER

```
public static final int CENTER
```

:   Possible alignment type. Indicates the elements should centered in
    the spaced provided.

    **See Also:**: `createParallelGroup(int)`, Constant Field Values

---


### BASELINE

```
public static final int BASELINE
```

:   Possible alignment type. Indicates the elements should aligned along
    their baseline.

    **See Also:**: `createParallelGroup(int)`, Constant Field Values

---


### DEFAULT\_SIZE

```
public static final int DEFAULT_SIZE
```

:   Possible value for the add methods that takes a Component.
    Indicates the size from the component should be used.

    **See Also:**: Constant Field Values

---


### PREFERRED\_SIZE

```
public static final int PREFERRED_SIZE
```

:   Possible value for the add methods that takes a Component.
    Indicates the preferred size should be used.

    **See Also:**: Constant Field Values


**Constructor Detail**

### GroupLayout

```
public GroupLayout(java.awt.Container host)
```

:   Creates a GroupLayout for the specified JComponent.

    **Parameters:**: `host` - the Container to layout **Throws:**: `java.lang.IllegalArgumentException` - if host is null


**Method Detail**

### setHonorsVisibility

```
public void setHonorsVisibility(boolean honorsVisibility)
```

:   Sets whether component visiblity is considered when sizing and
    positioning components. A value of `true` indicates that
    non-visible components should not be treated as part of the
    layout. A value of `false` indicates that components should be
    positioned and sized regardless of visibility.

    A value of `false` is useful when the visibility of components
    is dynamically adjusted and you don't want surrounding components and
    the sizing to change.

    The specified value is used for components that do not have an
    explicit visibility specified.

    The default is `true`.

    :   **Parameters:**: `honorsVisibility` - whether component visiblity is considered when sizing and positioning components **See Also:**: `setHonorsVisibility(Component,Boolean)`

---


### getHonorsVisibility

```
public boolean getHonorsVisibility()
```

:   Returns whether component visiblity is considered when sizing and
    positioning components.

    :   **Returns:**: whether component visiblity is considered when sizing and positioning components

---


### setHonorsVisibility

```
public void setHonorsVisibility(java.awt.Component component,
                                java.lang.Boolean honorsVisibility)
```

:   Sets whether the component's visiblity is considered for
    sizing and positioning. A value of `Boolean.TRUE`
    indicates that if `component` is not visible it should
    not be treated as part of the layout. A value of `false`
    indicates that `component` is positioned and sized
    regardless of it's visibility. A value of `null`
    indicates the value specified by the single argument method  `setHonorsVisibility` should be used.

    If `component` is not a child of the `Container` this
    `GroupLayout` is managing, it will be added to the
    `Container`.

    :   **Parameters:**: `component` - the component: `honorsVisibility` - whether `component`'s visiblity should be considered for sizing and positioning **Throws:**: `java.lang.IllegalArgumentException` - if `component` is `null` **See Also:**: `setHonorsVisibility(boolean)`

---


### toString

```
public java.lang.String toString()
```

:   Returns a textual description of this GroupLayout. The return value
    is intended for debugging purposes only.

    :   **Overrides:**: `toString` in class `java.lang.Object`
    :   **Returns:**: textual description of this GroupLayout

---


### setAutocreateGaps

```
public void setAutocreateGaps(boolean autocreatePadding)
```

:   Sets whether or not a gap between components
    should automatically be created. For example, if this is true
    and you add two components to a `SequentialGroup` a
    gap between the two will automatically be created. The default
    is false.

    :   **Parameters:**: `autocreatePadding` - whether or not to automatically created a gap between components and the container

---


### getAutocreateGaps

```
public boolean getAutocreateGaps()
```

:   Returns true if gaps between components are automatically be created.

    :   **Returns:**: true if gaps between components should automatically be created

---


### setAutocreateContainerGaps

```
public void setAutocreateContainerGaps(boolean autocreatePadding)
```

:   Sets whether or not gaps between the container and the first/last
    components should automatically be created. The default
    is false.

    :   **Parameters:**: `autocreatePadding` - whether or not to automatically create gaps between the container and first/last components.

---


### getAutocreateContainerGaps

```
public boolean getAutocreateContainerGaps()
```

:   Returns whether or not gaps between the container and the
    first/last components should automatically be created. The default
    is false.

    :   **Returns:**: whether or not the gaps between the container and the first/last components should automatically be created

---


### setHorizontalGroup

```
public void setHorizontalGroup(GroupLayout.Group group)
```

:   Sets the `Group` that is responsible for
    layout along the horizontal axis.

    :   **Parameters:**: `group` - `Group` responsible for layout along the horizontal axis **Throws:**: `java.lang.IllegalArgumentException` - if group is null

---


### getHorizontalGroup

```
public GroupLayout.Group getHorizontalGroup()
```

:   Returns the `Group` that is responsible for
    layout along the horizontal axis.

    :   **Returns:**: `ParallelGroup` responsible for layout along the horizontal axis.

---


### setVerticalGroup

```
public void setVerticalGroup(GroupLayout.Group group)
```

:   Sets the `Group` that is responsible for
    layout along the vertical axis.

    :   **Parameters:**: `group` - `Group` responsible for layout along the vertical axis. **Throws:**: `java.lang.IllegalArgumentException` - if group is null.

---


### getVerticalGroup

```
public GroupLayout.Group getVerticalGroup()
```

:   Returns the `ParallelGroup` that is responsible for
    layout along the vertical axis.

    :   **Returns:**: `ParallelGroup` responsible for layout along the vertical axis.

---


### createSequentialGroup

```
public GroupLayout.SequentialGroup createSequentialGroup()
```

:   Creates and returns a `SequentialGroup`.

    :   **Returns:**: a new `SequentialGroup`

---


### createParallelGroup

```
public GroupLayout.ParallelGroup createParallelGroup()
```

:   Creates and returns a `ParallelGroup` with a
    `LEADING` alignment. This is a cover method for the more
    general `createParallelGroup(int)` method.

    :   **Returns:**: a new ParallelGroup **See Also:**: `createParallelGroup(int)`

---


### createParallelGroup

```
public GroupLayout.ParallelGroup createParallelGroup(int alignment)
```

:   Creates and returns an `ParallelGroup`. The alignment
    specifies how children elements should be positioned when the
    the parallel group is given more space than necessary. For example,
    if a ParallelGroup with an alignment of TRAILING is given 100 pixels
    and a child only needs 50 pixels, the child will be positioned at the
    position 50.

    :   **Parameters:**: `alignment` - alignment for the elements of the Group, one of `LEADING`, `TRAILING`, `CENTER` or `BASELINE`. **Returns:**: a new `ParallelGroup` **Throws:**: `java.lang.IllegalArgumentException` - if alignment is not one of `LEADING`, `TRAILING`, `CENTER` or `BASELINE`

---


### createParallelGroup

```
public GroupLayout.ParallelGroup createParallelGroup(int alignment,
                                                     boolean resizable)
```

:   Creates and returns an `ParallelGroup`. The alignment
    specifies how children elements should be positioned when the
    the parallel group is given more space than necessary. For example,
    if a ParallelGroup with an alignment of TRAILING is given 100 pixels
    and a child only needs 50 pixels, the child will be positioned at the
    position 50.

    :   **Parameters:**: `alignment` - alignment for the elements of the Group, one of `LEADING`, `TRAILING`, `CENTER` or `BASELINE`.: `resizable` - whether or not the group is resizable. If the group is not resizable the min/max size will be the same as the preferred. **Returns:**: a new `ParallelGroup` **Throws:**: `java.lang.IllegalArgumentException` - if alignment is not one of `LEADING`, `TRAILING`, `CENTER` or `BASELINE`

---


### createBaselineGroup

```
public GroupLayout.ParallelGroup createBaselineGroup(boolean resizable,
                                                     boolean anchorBaselineToTop)
```

:   Creates and returns a `ParallelGroup` that aligns it's
    elements along the baseline.

    :   **Parameters:**: `resizable` - whether the group is resizable: `anchorBaselineToTop` - whether the baseline is anchored to the top or bottom of the group **See Also:**: `createBaselineGroup(boolean, boolean)`, `GroupLayout.ParallelGroup`

---


### linkSize

```
public void linkSize(java.awt.Component[] components)
```

:   Forces the set of components to have the same size.
    This can be used multiple times to force
    any number of components to share the same size.

    Linked Components are not be resizable.

    :   **Parameters:**: `components` - Components to force to have same size. **Throws:**: `java.lang.IllegalArgumentException` - if `components` is null, or contains null.

---


### linkSize

```
public void linkSize(java.awt.Component[] components,
                     int axis)
```

:   Forces the set of components to have the same size.
    This can be used multiple times to force
    any number of components to share the same size.

    Linked Components are not be resizable.

    :   **Parameters:**: `components` - Components to force to have same size.: `axis` - Axis to bind size, one of HORIZONTAL, VERTICAL or HORIZONTAL | VERTICAL **Throws:**: `java.lang.IllegalArgumentException` - if `components` is null, or contains null.: `java.lang.IllegalArgumentException` - if `axis` does not contain `HORIZONTAL` or `VERTICAL`

---


### replace

```
public void replace(java.awt.Component existingComponent,
                    java.awt.Component newComponent)
```

:   Removes an existing component replacing it with the specified component.

    :   **Parameters:**: `existingComponent` - the Component that should be removed and replaced with newComponent: `newComponent` - the Component to put in existingComponents place **Throws:**: `java.lang.IllegalArgumentException` - is either of the Components are null or if existingComponent is not being managed by this layout manager

---


### setLayoutStyle

```
public void setLayoutStyle(LayoutStyle layoutStyle)
```

:   Sets the LayoutStyle this GroupLayout is to use. A value of null can
    be used to indicate the shared instance of LayoutStyle should be used.

    :   **Parameters:**: `layoutStyle` - the LayoutStyle to use

---


### getLayoutStyle

```
public LayoutStyle getLayoutStyle()
```

:   Returns the LayoutStyle instance to use

    :   **Returns:**: the LayoutStyle instance to use

---


### addLayoutComponent

```
public void addLayoutComponent(java.lang.String name,
                               java.awt.Component component)
```

:   Notification that a `Component` has been added to
    the parent container. Developers should not invoke this method
    directly, instead you should use one of the `Group`
    methods to add a `Component`.

    :   **Specified by:**: `addLayoutComponent` in interface `java.awt.LayoutManager`
    :   **Parameters:**: `name` - the string to be associated with the component: `component` - the `Component` to be added

---


### removeLayoutComponent

```
public void removeLayoutComponent(java.awt.Component component)
```

:   Notification that a `Component` has been removed from
    the parent container. You should not invoke this method
    directly, instead invoke `remove` on the parent
    `Container`.

    :   **Specified by:**: `removeLayoutComponent` in interface `java.awt.LayoutManager`
    :   **Parameters:**: `component` - the component to be removed **See Also:**: `Component.remove(java.awt.MenuComponent)`

---


### preferredLayoutSize

```
public java.awt.Dimension preferredLayoutSize(java.awt.Container parent)
```

:   Returns the preferred size for the specified container.

    :   **Specified by:**: `preferredLayoutSize` in interface `java.awt.LayoutManager`
    :   **Parameters:**: `parent` - the container to return size for **Throws:**: `java.lang.IllegalArgumentException` - if `parent` is not the same `Container` that this was created with: `java.lang.IllegalStateException` - if any of the components added to this layout are not in both a horizontal and vertical group **See Also:**: `Container.getPreferredSize()`

---


### minimumLayoutSize

```
public java.awt.Dimension minimumLayoutSize(java.awt.Container parent)
```

:   Returns the minimum size for the specified container.

    :   **Specified by:**: `minimumLayoutSize` in interface `java.awt.LayoutManager`
    :   **Parameters:**: `parent` - the container to return size for **Throws:**: `java.lang.IllegalArgumentException` - if `parent` is not the same `Container` that this was created with: `java.lang.IllegalStateException` - if any of the components added to this layout are not in both a horizontal and vertical group **See Also:**: `Container.getMinimumSize()`

---


### layoutContainer

```
public void layoutContainer(java.awt.Container parent)
```

:   Lays out the specified container.

    :   **Specified by:**: `layoutContainer` in interface `java.awt.LayoutManager`
    :   **Parameters:**: `parent` - the container to be laid out **Throws:**: `java.lang.IllegalStateException` - if any of the components added to this layout are not in both a horizontal and vertical group

---


### addLayoutComponent

```
public void addLayoutComponent(java.awt.Component component,
                               java.lang.Object constraints)
```

:   Notification that a `Component` has been added to
    the parent container. You should not invoke this method
    directly, instead you should use one of the `Group`
    methods to add a `Component`.

    :   **Specified by:**: `addLayoutComponent` in interface `java.awt.LayoutManager2`
    :   **Parameters:**: `component` - The component added: `constraints` - Description of where to place the component.

---


### maximumLayoutSize

```
public java.awt.Dimension maximumLayoutSize(java.awt.Container parent)
```

:   Returns the maximum size for the specified container.

    :   **Specified by:**: `maximumLayoutSize` in interface `java.awt.LayoutManager2`
    :   **Parameters:**: `parent` - the container to return size for **Throws:**: `java.lang.IllegalArgumentException` - if `parent` is not the same `Container` that this was created with: `java.lang.IllegalStateException` - if any of the components added to this layout are not in both a horizontal and vertical group **See Also:**: `Container.getMaximumSize()`

---


### getLayoutAlignmentX

```
public float getLayoutAlignmentX(java.awt.Container parent)
```

:   Returns the alignment along the x axis. This specifies how
    the component would like to be aligned relative to other
    components. The value should be a number between 0 and 1
    where 0 represents alignment along the origin, 1 is aligned
    the furthest away from the origin, 0.5 is centered, etc.

    :   **Specified by:**: `getLayoutAlignmentX` in interface `java.awt.LayoutManager2`
    :   **Parameters:**: `parent` - Container hosting this LayoutManager **Returns:**: alignment **Throws:**: `java.lang.IllegalArgumentException` - if `parent` is not the same `Container` that this was created with

---


### getLayoutAlignmentY

```
public float getLayoutAlignmentY(java.awt.Container parent)
```

:   Returns the alignment along the y axis. This specifies how
    the component would like to be aligned relative to other
    components. The value should be a number between 0 and 1
    where 0 represents alignment along the origin, 1 is aligned
    the furthest away from the origin, 0.5 is centered, etc.

    :   **Specified by:**: `getLayoutAlignmentY` in interface `java.awt.LayoutManager2`
    :   **Parameters:**: `parent` - Container hosting this LayoutManager **Returns:**: alignment **Throws:**: `java.lang.IllegalArgumentException` - if `parent` is not the same `Container` that this was created with

---


### invalidateLayout

```
public void invalidateLayout(java.awt.Container parent)
```

:   Invalidates the layout, indicating that if the layout manager
    has cached information it should be discarded.

    :   **Specified by:**: `invalidateLayout` in interface `java.awt.LayoutManager2`
    :   **Parameters:**: `parent` - Container hosting this LayoutManager **Throws:**: `java.lang.IllegalArgumentException` - if `parent` is not the same `Container` that this was created with


---


|  |  |  |
| --- | --- | --- |
| **Package** \| **Class** \| **Use** \| **Tree** \| **Deprecated** \| **Index** \| **Help** | |  |
| **PREV CLASS**   **NEXT CLASS** | **FRAMES**    **NO FRAMES**     **All Classes** |  |
| SUMMARY: NESTED \| FIELD \| CONSTR \| METHOD | DETAIL: FIELD \| CONSTR \| METHOD |  |


---
