# Supplementary material for: RGG: A general GUI Framework for R scripts
Source: BMC Bioinformatics. 2009 Mar 2;10:74. doi: 10.1186/1471-2105-10-74 (PMC2653488; doi:10.1186/1471-2105-10-74)
Supplement: Additional file 2 — RGG runner for Windows/Linux. Software to load and execute rgg files (Windows and Linux). [file 1471-2105-10-74-S2.zip › rggrunner/platform8/docs/swing-layout-1.0.3-doc.zip!/org/jdesktop/layout/LayoutStyle.html]

LayoutStyle


|  |  |  |
| --- | --- | --- |
| **Package** \| **Class** \| **Use** \| **Tree** \| **Deprecated** \| **Index** \| **Help** | |  |
| **PREV CLASS**   NEXT CLASS | **FRAMES**    **NO FRAMES**     **All Classes** |  |
| SUMMARY: NESTED \| FIELD \| CONSTR \| METHOD | DETAIL: FIELD \| CONSTR \| METHOD |  |


---


## org.jdesktop.layout Class LayoutStyle

```
java.lang.Object
  org.jdesktop.layout.LayoutStyle
```

---

``` public class LayoutStyle extends java.lang.Object ```

LayoutStyle is used to determine how much space to place between components
during layout. LayoutStyle can be obtained for two components, or for
a component relative to an edge of a parent container. The amount of
space can vary depending upon whether or not the components are
logically grouped together (`RELATED`).

This class is primarily useful for JREs prior to 1.6. In 1.6 API for this
was added to Swing. When run on a JRE of 1.6 or greater this will call into
the appropriate methods in Swing.

---

| **Field Summary** | |
| --- | --- |
| `static int` | `INDENT`             Possible argument to getPreferredGap. |
| `static int` | `RELATED`             Possible argument to getPreferredGap. |
| `static int` | `UNRELATED`             Possible argument to getPreferredGap. |


**Constructor Summary**

`LayoutStyle()`


| **Method Summary** | |
| --- | --- |
| `int` | `getContainerGap(javax.swing.JComponent component, int position, java.awt.Container parent)`             Returns the amount of space to position a component inside its parent. |
| `int` | `getPreferredGap(javax.swing.JComponent component1, javax.swing.JComponent component2, int type, int position, java.awt.Container parent)`             Returns the amount of space to use between two components. |
| `static LayoutStyle` | `getSharedInstance()`             Factory methods for obtaining the current `LayoutStyle` object appropriate for the current look and feel. |
| `static void` | `setSharedInstance(LayoutStyle layoutStyle)`             Sets the LayoutStyle instance to use for this look and feel. |

**Methods inherited from class java.lang.Object**

`clone, equals, finalize, getClass, hashCode, notify, notifyAll, toString, wait, wait, wait`

**Field Detail**

### RELATED

```
public static final int RELATED
```

:   Possible argument to getPreferredGap. Used to indicate the two componets
    are grouped together.

    **See Also:**: Constant Field Values

---


### UNRELATED

```
public static final int UNRELATED
```

:   Possible argument to getPreferredGap. Used to indicate the two componets
    are not grouped together.

    **See Also:**: Constant Field Values

---


### INDENT

```
public static final int INDENT
```

:   Possible argument to getPreferredGap. Used to indicate the distance
    to indent a component is being requested. To visually indicate
    a set of related components they will often times be horizontally
    indented, the `INDENT` constant for this.
    For example, to indent a check box relative to a label use this
    constant to `getPreferredGap`.

    **See Also:**: Constant Field Values


**Constructor Detail**

### LayoutStyle

```
public LayoutStyle()
```


**Method Detail**

### setSharedInstance

```
public static void setSharedInstance(LayoutStyle layoutStyle)
```

:   Sets the LayoutStyle instance to use for this look and feel.
    You generally don't need to invoke this, instead use the getter which
    will return the LayoutStyle appropriate for the current look and feel.

    :   **Parameters:**: `layoutStyle` - the LayoutStyle to use; a value of null indicates the default should be used

---


### getSharedInstance

```
public static LayoutStyle getSharedInstance()
```

:   Factory methods for obtaining the current `LayoutStyle`
    object appropriate for the current look and feel.

    :   **Returns:**: the current LayoutStyle instance

---


### getPreferredGap

```
public int getPreferredGap(javax.swing.JComponent component1,
                           javax.swing.JComponent component2,
                           int type,
                           int position,
                           java.awt.Container parent)
```

:   Returns the amount of space to use between two components.
    The return value indicates the distance to place
    `component2` relative to `component1`.
    For example, the following returns the amount of space to place
    between `component2` and `component1`
    when `component2` is placed vertically above
    `component1`:

    ```
       int gap = getPreferredGap(component1, component2,
                                 LayoutStyle.RELATED,
                                 SwingConstants.NORTH, parent);
    ```

    The `type` parameter indicates the type
    of gap being requested. It can be one of the following values:

    |  |  |  |  |  |  |
    | --- | --- | --- | --- | --- | --- |
    | `RELATED` If the two components will be contained in the same parent and are showing similar logically related items, use `RELATED`.| `UNRELATED` If the two components will be contained in the same parent but show logically unrelated items use `UNRELATED`.| `INDENT` Used to obtain the preferred distance to indent a component relative to another. For example, if you want to horizontally indent a JCheckBox relative to a JLabel use `INDENT`. This is only useful for the horizontal axis. | | | | | |

    It's important to note that some look and feels may not distinguish
    between `RELATED` and `UNRELATED`.

    The return value is not intended to take into account the
    current size and position of `component2` or
    `component1`. The return value may take into
    consideration various properties of the components. For
    example, the space may vary based on font size, or the preferred
    size of the component.

    :   **Parameters:**: `component1` - the `JComponent` `component2` is being placed relative to: `component2` - the `JComponent` being placed: `type` - how the two components are being placed: `position` - the position `component2` is being placed relative to `component1`; one of `SwingConstants.NORTH`, `SwingConstants.SOUTH`, `SwingConstants.EAST` or `SwingConstants.WEST`: `parent` - the parent of `component2`; this may differ from the actual parent and may be null **Returns:**: the amount of space to place between the two components **Throws:**: `java.lang.IllegalArgumentException` - if `position` is not one of `SwingConstants.NORTH`, `SwingConstants.SOUTH`, `SwingConstants.EAST` or `SwingConstants.WEST`; `type` not one of `INDENT`, `RELATED` or `UNRELATED`; or `component1` or `component2` is null

---


### getContainerGap

```
public int getContainerGap(javax.swing.JComponent component,
                           int position,
                           java.awt.Container parent)
```

:   Returns the amount of space to position a component inside its
    parent.

    :   **Parameters:**: `component` - the `Component` being positioned: `position` - the position `component` is being placed relative to its parent; one of `SwingConstants.NORTH`, `SwingConstants.SOUTH`, `SwingConstants.EAST` or `SwingConstants.WEST`: `parent` - the parent of `component`; this may differ from the actual parent and may be null **Returns:**: the amount of space to place between the component and specified edge **Throws:**: `java.lang.IllegalArgumentException` - if `position` is not one of `SwingConstants.NORTH`, `SwingConstants.SOUTH`, `SwingConstants.EAST` or `SwingConstants.WEST`; or `component` is null


---


|  |  |  |
| --- | --- | --- |
| **Package** \| **Class** \| **Use** \| **Tree** \| **Deprecated** \| **Index** \| **Help** | |  |
| **PREV CLASS**   NEXT CLASS | **FRAMES**    **NO FRAMES**     **All Classes** |  |
| SUMMARY: NESTED \| FIELD \| CONSTR \| METHOD | DETAIL: FIELD \| CONSTR \| METHOD |  |


---
