# Supplementary material for: RGG: A general GUI Framework for R scripts
Source: BMC Bioinformatics. 2009 Mar 2;10:74. doi: 10.1186/1471-2105-10-74 (PMC2653488; doi:10.1186/1471-2105-10-74)
Supplement: Additional file 2 — RGG runner for Windows/Linux. Software to load and execute rgg files (Windows and Linux). [file 1471-2105-10-74-S2.zip › rggrunner/platform8/docs/swing-layout-1.0.3-doc.zip!/org/jdesktop/layout/class-use/GroupLayout.Group.html]

Uses of Class org.jdesktop.layout.GroupLayout.Group


|  |  |  |
| --- | --- | --- |
| **Package** \| **Class** \| **Use** \| **Tree** \| **Deprecated** \| **Index** \| **Help** | |  |
| PREV   NEXT | **FRAMES**    **NO FRAMES**     **All Classes** |  |


---


## **Uses of Class org.jdesktop.layout.GroupLayout.Group**


Uses of GroupLayout.Group in org.jdesktop.layout

| Subclasses of GroupLayout.Group in org.jdesktop.layout | |
| --- | --- |
| `class` | `GroupLayout.ParallelGroup`             A `Group` that lays out its elements on top of each other. |
| `class` | `GroupLayout.SequentialGroup`             A `Group` that lays out its elements sequentially, one after another. |

| Methods in org.jdesktop.layout that return GroupLayout.Group | |
| --- | --- |
| `GroupLayout.Group` | `GroupLayout.getHorizontalGroup()`             Returns the `Group` that is responsible for layout along the horizontal axis. |
| `GroupLayout.Group` | `GroupLayout.getHorizontalGroup()`             Returns the `Group` that is responsible for layout along the horizontal axis. |
| `GroupLayout.Group` | `GroupLayout.getVerticalGroup()`             Returns the `ParallelGroup` that is responsible for layout along the vertical axis. |
| `GroupLayout.Group` | `GroupLayout.getVerticalGroup()`             Returns the `ParallelGroup` that is responsible for layout along the vertical axis. |

| Methods in org.jdesktop.layout with parameters of type GroupLayout.Group | |
| --- | --- |
| `GroupLayout.SequentialGroup` | `GroupLayout.SequentialGroup.add(boolean useAsBaseline, GroupLayout.Group group)`             Adds a `Group` to this `Group`. |
| `GroupLayout.SequentialGroup` | `GroupLayout.SequentialGroup.add(boolean useAsBaseline, GroupLayout.Group group)`             Adds a `Group` to this `Group`. |
| `GroupLayout.SequentialGroup` | `GroupLayout.SequentialGroup.add(GroupLayout.Group group)`             Adds the specified `Group` to this `SequentialGroup` |
| `GroupLayout.ParallelGroup` | `GroupLayout.ParallelGroup.add(GroupLayout.Group group)`             Adds the specified `Group`. |
| `GroupLayout.SequentialGroup` | `GroupLayout.SequentialGroup.add(GroupLayout.Group group)`             Adds the specified `Group` to this `SequentialGroup` |
| `GroupLayout.ParallelGroup` | `GroupLayout.ParallelGroup.add(GroupLayout.Group group)`             Adds the specified `Group`. |
| `GroupLayout.ParallelGroup` | `GroupLayout.ParallelGroup.add(int alignment, GroupLayout.Group group)`             Adds the specified `Group` as a child of this group. |
| `GroupLayout.ParallelGroup` | `GroupLayout.ParallelGroup.add(int alignment, GroupLayout.Group group)`             Adds the specified `Group` as a child of this group. |
| `void` | `GroupLayout.setHorizontalGroup(GroupLayout.Group group)`             Sets the `Group` that is responsible for layout along the horizontal axis. |
| `void` | `GroupLayout.setHorizontalGroup(GroupLayout.Group group)`             Sets the `Group` that is responsible for layout along the horizontal axis. |
| `void` | `GroupLayout.setVerticalGroup(GroupLayout.Group group)`             Sets the `Group` that is responsible for layout along the vertical axis. |
| `void` | `GroupLayout.setVerticalGroup(GroupLayout.Group group)`             Sets the `Group` that is responsible for layout along the vertical axis. |

---


|  |  |  |
| --- | --- | --- |
| **Package** \| **Class** \| **Use** \| **Tree** \| **Deprecated** \| **Index** \| **Help** | |  |
| PREV   NEXT | **FRAMES**    **NO FRAMES**     **All Classes** |  |


---
