# Supplementary material for: RGG: A general GUI Framework for R scripts
Source: BMC Bioinformatics. 2009 Mar 2;10:74. doi: 10.1186/1471-2105-10-74 (PMC2653488; doi:10.1186/1471-2105-10-74)
Supplement: Additional file 2 — RGG runner for Windows/Linux. Software to load and execute rgg files (Windows and Linux). [file 1471-2105-10-74-S2.zip › rggrunner/platform8/docs/swing-layout-1.0.3-doc.zip!/org/jdesktop/layout/class-use/GroupLayout.ParallelGroup.html]

Uses of Class org.jdesktop.layout.GroupLayout.ParallelGroup


|  |  |  |
| --- | --- | --- |
| **Package** \| **Class** \| **Use** \| **Tree** \| **Deprecated** \| **Index** \| **Help** | |  |
| PREV   NEXT | **FRAMES**    **NO FRAMES**     **All Classes** |  |


---


## **Uses of Class org.jdesktop.layout.GroupLayout.ParallelGroup**


Uses of GroupLayout.ParallelGroup in org.jdesktop.layout

| Methods in org.jdesktop.layout that return GroupLayout.ParallelGroup | |
| --- | --- |
| `GroupLayout.ParallelGroup` | `GroupLayout.ParallelGroup.add(java.awt.Component component)`             Adds the specified Component. |
| `GroupLayout.ParallelGroup` | `GroupLayout.ParallelGroup.add(java.awt.Component component)`             Adds the specified Component. |
| `GroupLayout.ParallelGroup` | `GroupLayout.ParallelGroup.add(java.awt.Component component, int min, int pref, int max)`             Adds the specified `Component`. |
| `GroupLayout.ParallelGroup` | `GroupLayout.ParallelGroup.add(java.awt.Component component, int min, int pref, int max)`             Adds the specified `Component`. |
| `GroupLayout.ParallelGroup` | `GroupLayout.ParallelGroup.add(GroupLayout.Group group)`             Adds the specified `Group`. |
| `GroupLayout.ParallelGroup` | `GroupLayout.ParallelGroup.add(GroupLayout.Group group)`             Adds the specified `Group`. |
| `GroupLayout.ParallelGroup` | `GroupLayout.ParallelGroup.add(int pref)`             Adds a rigid gap. |
| `GroupLayout.ParallelGroup` | `GroupLayout.ParallelGroup.add(int pref)`             Adds a rigid gap. |
| `GroupLayout.ParallelGroup` | `GroupLayout.ParallelGroup.add(int alignment, java.awt.Component component)`             Adds the specified Component. |
| `GroupLayout.ParallelGroup` | `GroupLayout.ParallelGroup.add(int alignment, java.awt.Component component)`             Adds the specified Component. |
| `GroupLayout.ParallelGroup` | `GroupLayout.ParallelGroup.add(int alignment, java.awt.Component component, int min, int pref, int max)`             Adds the specified `Component`. |
| `GroupLayout.ParallelGroup` | `GroupLayout.ParallelGroup.add(int alignment, java.awt.Component component, int min, int pref, int max)`             Adds the specified `Component`. |
| `GroupLayout.ParallelGroup` | `GroupLayout.ParallelGroup.add(int alignment, GroupLayout.Group group)`             Adds the specified `Group` as a child of this group. |
| `GroupLayout.ParallelGroup` | `GroupLayout.ParallelGroup.add(int alignment, GroupLayout.Group group)`             Adds the specified `Group` as a child of this group. |
| `GroupLayout.ParallelGroup` | `GroupLayout.ParallelGroup.add(int min, int pref, int max)`             Adds a gap with the specified size. |
| `GroupLayout.ParallelGroup` | `GroupLayout.ParallelGroup.add(int min, int pref, int max)`             Adds a gap with the specified size. |
| `GroupLayout.ParallelGroup` | `GroupLayout.createBaselineGroup(boolean resizable, boolean anchorBaselineToTop)`             Creates and returns a `ParallelGroup` that aligns it's elements along the baseline. |
| `GroupLayout.ParallelGroup` | `GroupLayout.createBaselineGroup(boolean resizable, boolean anchorBaselineToTop)`             Creates and returns a `ParallelGroup` that aligns it's elements along the baseline. |
| `GroupLayout.ParallelGroup` | `GroupLayout.createParallelGroup()`             Creates and returns a `ParallelGroup` with a `LEADING` alignment. |
| `GroupLayout.ParallelGroup` | `GroupLayout.createParallelGroup()`             Creates and returns a `ParallelGroup` with a `LEADING` alignment. |
| `GroupLayout.ParallelGroup` | `GroupLayout.createParallelGroup(int alignment)`             Creates and returns an `ParallelGroup`. |
| `GroupLayout.ParallelGroup` | `GroupLayout.createParallelGroup(int alignment)`             Creates and returns an `ParallelGroup`. |
| `GroupLayout.ParallelGroup` | `GroupLayout.createParallelGroup(int alignment, boolean resizable)`             Creates and returns an `ParallelGroup`. |
| `GroupLayout.ParallelGroup` | `GroupLayout.createParallelGroup(int alignment, boolean resizable)`             Creates and returns an `ParallelGroup`. |

---


|  |  |  |
| --- | --- | --- |
| **Package** \| **Class** \| **Use** \| **Tree** \| **Deprecated** \| **Index** \| **Help** | |  |
| PREV   NEXT | **FRAMES**    **NO FRAMES**     **All Classes** |  |


---
