# Supplementary material for: RGG: A general GUI Framework for R scripts
Source: BMC Bioinformatics. 2009 Mar 2;10:74. doi: 10.1186/1471-2105-10-74 (PMC2653488; doi:10.1186/1471-2105-10-74)
Supplement: Additional file 2 — RGG runner for Windows/Linux. Software to load and execute rgg files (Windows and Linux). [file 1471-2105-10-74-S2.zip › rggrunner/platform8/docs/swing-layout-1.0.3-doc.zip!/org/jdesktop/layout/class-use/GroupLayout.SequentialGroup.html]

Uses of Class org.jdesktop.layout.GroupLayout.SequentialGroup


|  |  |  |
| --- | --- | --- |
| **Package** \| **Class** \| **Use** \| **Tree** \| **Deprecated** \| **Index** \| **Help** | |  |
| PREV   NEXT | **FRAMES**    **NO FRAMES**     **All Classes** |  |


---


## **Uses of Class org.jdesktop.layout.GroupLayout.SequentialGroup**


Uses of GroupLayout.SequentialGroup in org.jdesktop.layout

| Methods in org.jdesktop.layout that return GroupLayout.SequentialGroup | |
| --- | --- |
| `GroupLayout.SequentialGroup` | `GroupLayout.SequentialGroup.add(boolean useAsBaseline, java.awt.Component component)`             Adds a `Component` to this `Group`. |
| `GroupLayout.SequentialGroup` | `GroupLayout.SequentialGroup.add(boolean useAsBaseline, java.awt.Component component)`             Adds a `Component` to this `Group`. |
| `GroupLayout.SequentialGroup` | `GroupLayout.SequentialGroup.add(boolean useAsBaseline, java.awt.Component component, int min, int pref, int max)`             Adds a `Component` to this `Group` with the specified size. |
| `GroupLayout.SequentialGroup` | `GroupLayout.SequentialGroup.add(boolean useAsBaseline, java.awt.Component component, int min, int pref, int max)`             Adds a `Component` to this `Group` with the specified size. |
| `GroupLayout.SequentialGroup` | `GroupLayout.SequentialGroup.add(boolean useAsBaseline, GroupLayout.Group group)`             Adds a `Group` to this `Group`. |
| `GroupLayout.SequentialGroup` | `GroupLayout.SequentialGroup.add(boolean useAsBaseline, GroupLayout.Group group)`             Adds a `Group` to this `Group`. |
| `GroupLayout.SequentialGroup` | `GroupLayout.SequentialGroup.add(java.awt.Component component)`             Adds the specified Component. |
| `GroupLayout.SequentialGroup` | `GroupLayout.SequentialGroup.add(java.awt.Component component)`             Adds the specified Component. |
| `GroupLayout.SequentialGroup` | `GroupLayout.SequentialGroup.add(java.awt.Component component, int min, int pref, int max)`             Adds the specified `Component`. |
| `GroupLayout.SequentialGroup` | `GroupLayout.SequentialGroup.add(java.awt.Component component, int min, int pref, int max)`             Adds the specified `Component`. |
| `GroupLayout.SequentialGroup` | `GroupLayout.SequentialGroup.add(GroupLayout.Group group)`             Adds the specified `Group` to this `SequentialGroup` |
| `GroupLayout.SequentialGroup` | `GroupLayout.SequentialGroup.add(GroupLayout.Group group)`             Adds the specified `Group` to this `SequentialGroup` |
| `GroupLayout.SequentialGroup` | `GroupLayout.SequentialGroup.add(int pref)`             Adds a rigid gap. |
| `GroupLayout.SequentialGroup` | `GroupLayout.SequentialGroup.add(int pref)`             Adds a rigid gap. |
| `GroupLayout.SequentialGroup` | `GroupLayout.SequentialGroup.add(int min, int pref, int max)`             Adds a gap with the specified size. |
| `GroupLayout.SequentialGroup` | `GroupLayout.SequentialGroup.add(int min, int pref, int max)`             Adds a gap with the specified size. |
| `GroupLayout.SequentialGroup` | `GroupLayout.SequentialGroup.addContainerGap()`             Adds an element representing the preferred gap between one edge of the container and the next/previous Component. |
| `GroupLayout.SequentialGroup` | `GroupLayout.SequentialGroup.addContainerGap()`             Adds an element representing the preferred gap between one edge of the container and the next/previous Component. |
| `GroupLayout.SequentialGroup` | `GroupLayout.SequentialGroup.addContainerGap(int pref, int max)`             Adds an element representing the preferred gap between one edge of the container and the next/previous Component. |
| `GroupLayout.SequentialGroup` | `GroupLayout.SequentialGroup.addContainerGap(int pref, int max)`             Adds an element representing the preferred gap between one edge of the container and the next/previous Component. |
| `GroupLayout.SequentialGroup` | `GroupLayout.SequentialGroup.addPreferredGap(int type)`             Adds an element representing the preferred gap between the nearest components. |
| `GroupLayout.SequentialGroup` | `GroupLayout.SequentialGroup.addPreferredGap(int type)`             Adds an element representing the preferred gap between the nearest components. |
| `GroupLayout.SequentialGroup` | `GroupLayout.SequentialGroup.addPreferredGap(int type, int pref, int max)`             Adds an element for the preferred gap between the nearest components. |
| `GroupLayout.SequentialGroup` | `GroupLayout.SequentialGroup.addPreferredGap(int type, int pref, int max)`             Adds an element for the preferred gap between the nearest components. |
| `GroupLayout.SequentialGroup` | `GroupLayout.SequentialGroup.addPreferredGap(javax.swing.JComponent comp1, javax.swing.JComponent comp2, int type)`             Adds an element representing the preferred gap between the two components. |
| `GroupLayout.SequentialGroup` | `GroupLayout.SequentialGroup.addPreferredGap(javax.swing.JComponent comp1, javax.swing.JComponent comp2, int type)`             Adds an element representing the preferred gap between the two components. |
| `GroupLayout.SequentialGroup` | `GroupLayout.SequentialGroup.addPreferredGap(javax.swing.JComponent comp1, javax.swing.JComponent comp2, int type, boolean canGrow)`             Adds an element representing the preferred gap between the two components. |
| `GroupLayout.SequentialGroup` | `GroupLayout.SequentialGroup.addPreferredGap(javax.swing.JComponent comp1, javax.swing.JComponent comp2, int type, boolean canGrow)`             Adds an element representing the preferred gap between the two components. |
| `GroupLayout.SequentialGroup` | `GroupLayout.createSequentialGroup()`             Creates and returns a `SequentialGroup`. |
| `GroupLayout.SequentialGroup` | `GroupLayout.createSequentialGroup()`             Creates and returns a `SequentialGroup`. |

---


|  |  |  |
| --- | --- | --- |
| **Package** \| **Class** \| **Use** \| **Tree** \| **Deprecated** \| **Index** \| **Help** | |  |
| PREV   NEXT | **FRAMES**    **NO FRAMES**     **All Classes** |  |


---
