# Supplementary material for: RGG: A general GUI Framework for R scripts
Source: BMC Bioinformatics. 2009 Mar 2;10:74. doi: 10.1186/1471-2105-10-74 (PMC2653488; doi:10.1186/1471-2105-10-74)
Supplement: Additional file 2 — RGG runner for Windows/Linux. Software to load and execute rgg files (Windows and Linux). [file 1471-2105-10-74-S2.zip › rggrunner/platform8/docs/swing-layout-1.0.3-doc.zip!/org/jdesktop/layout/class-use/LayoutStyle.html]

Uses of Class org.jdesktop.layout.LayoutStyle


|  |  |  |
| --- | --- | --- |
| **Package** \| **Class** \| **Use** \| **Tree** \| **Deprecated** \| **Index** \| **Help** | |  |
| PREV   NEXT | **FRAMES**    **NO FRAMES**     **All Classes** |  |


---


## **Uses of Class org.jdesktop.layout.LayoutStyle**


Uses of LayoutStyle in org.jdesktop.layout

| Methods in org.jdesktop.layout that return LayoutStyle | |
| --- | --- |
| `LayoutStyle` | `GroupLayout.getLayoutStyle()`             Returns the LayoutStyle instance to use |
| `LayoutStyle` | `GroupLayout.getLayoutStyle()`             Returns the LayoutStyle instance to use |
| `static LayoutStyle` | `LayoutStyle.getSharedInstance()`             Factory methods for obtaining the current `LayoutStyle` object appropriate for the current look and feel. |
| `static LayoutStyle` | `LayoutStyle.getSharedInstance()`             Factory methods for obtaining the current `LayoutStyle` object appropriate for the current look and feel. |

| Methods in org.jdesktop.layout with parameters of type LayoutStyle | |
| --- | --- |
| `void` | `GroupLayout.setLayoutStyle(LayoutStyle layoutStyle)`             Sets the LayoutStyle this GroupLayout is to use. |
| `void` | `GroupLayout.setLayoutStyle(LayoutStyle layoutStyle)`             Sets the LayoutStyle this GroupLayout is to use. |
| `static void` | `LayoutStyle.setSharedInstance(LayoutStyle layoutStyle)`             Sets the LayoutStyle instance to use for this look and feel. |
| `static void` | `LayoutStyle.setSharedInstance(LayoutStyle layoutStyle)`             Sets the LayoutStyle instance to use for this look and feel. |

---


|  |  |  |
| --- | --- | --- |
| **Package** \| **Class** \| **Use** \| **Tree** \| **Deprecated** \| **Index** \| **Help** | |  |
| PREV   NEXT | **FRAMES**    **NO FRAMES**     **All Classes** |  |


---
