# Supplementary material for: RGG: A general GUI Framework for R scripts
Source: BMC Bioinformatics. 2009 Mar 2;10:74. doi: 10.1186/1471-2105-10-74 (PMC2653488; doi:10.1186/1471-2105-10-74)
Supplement: Additional file 2 — RGG runner for Windows/Linux. Software to load and execute rgg files (Windows and Linux). [file 1471-2105-10-74-S2.zip › rggrunner/platform8/docs/swing-layout-1.0.3-doc.zip!/org/jdesktop/layout/package-summary.html]

org.jdesktop.layout


|  |  |  |
| --- | --- | --- |
| **Package** \| Class \| **Use** \| **Tree** \| **Deprecated** \| **Index** \| **Help** | |  |
| PREV PACKAGE   NEXT PACKAGE | **FRAMES**    **NO FRAMES**     **All Classes** |  |


---

## Package org.jdesktop.layout

| **Class Summary** | |
| --- | --- |
| **Baseline** | Convenience class that can be used to determine the baseline of a particular component. |
| **GroupLayout** | GroupLayout is a LayoutManager that hierarchically groups components to achieve common, and not so common, layouts. |
| **LayoutStyle** | LayoutStyle is used to determine how much space to place between components during layout. |

---


|  |  |  |
| --- | --- | --- |
| **Package** \| Class \| **Use** \| **Tree** \| **Deprecated** \| **Index** \| **Help** | |  |
| PREV PACKAGE   NEXT PACKAGE | **FRAMES**    **NO FRAMES**     **All Classes** |  |


---
