# Supplementary material for: RGG: A general GUI Framework for R scripts
Source: BMC Bioinformatics. 2009 Mar 2;10:74. doi: 10.1186/1471-2105-10-74 (PMC2653488; doi:10.1186/1471-2105-10-74)
Supplement: Additional file 2 — RGG runner for Windows/Linux. Software to load and execute rgg files (Windows and Linux). [file 1471-2105-10-74-S2.zip › rggrunner/platform8/docs/swing-layout-1.0.3-doc.zip!/org/jdesktop/layout/package-use.html]

Uses of Package org.jdesktop.layout


|  |  |  |
| --- | --- | --- |
| **Package** \| Class \| **Use** \| **Tree** \| **Deprecated** \| **Index** \| **Help** | |  |
| PREV   NEXT | **FRAMES**    **NO FRAMES**     **All Classes** |  |


---


## **Uses of Package org.jdesktop.layout**


Classes in org.jdesktop.layout used by org.jdesktop.layout

****GroupLayout.Group****
  
          Group provides for commonality between the two types of operations
supported by `GroupLayout`: laying out components one
after another (`SequentialGroup`) or layout on top
of each other (`ParallelGroup`).

****GroupLayout.ParallelGroup****
  
          A `Group` that lays out its elements on top of each
other.

****GroupLayout.SequentialGroup****
  
          A `Group` that lays out its elements sequentially, one
after another.

****LayoutStyle****
  
          LayoutStyle is used to determine how much space to place between components
during layout.

---


|  |  |  |
| --- | --- | --- |
| **Package** \| Class \| **Use** \| **Tree** \| **Deprecated** \| **Index** \| **Help** | |  |
| PREV   NEXT | **FRAMES**    **NO FRAMES**     **All Classes** |  |


---
