# Supplementary material for: RGG: A general GUI Framework for R scripts
Source: BMC Bioinformatics. 2009 Mar 2;10:74. doi: 10.1186/1471-2105-10-74 (PMC2653488; doi:10.1186/1471-2105-10-74)
Supplement: Additional file 2 — RGG runner for Windows/Linux. Software to load and execute rgg files (Windows and Linux). [file 1471-2105-10-74-S2.zip › rggrunner/platform8/docs/swing-layout-1.0.3-doc.zip!/overview-tree.html]

Class Hierarchy


|  |  |  |
| --- | --- | --- |
| **Package** \| Class \| Use \| **Tree** \| **Deprecated** \| **Index** \| **Help** | |  |
| PREV   NEXT | **FRAMES**    **NO FRAMES**     **All Classes** |  |


---


## Hierarchy For All Packages

**Package Hierarchies:**: org.jdesktop.layout

---

## Class Hierarchy

- java.lang.Object
  - org.jdesktop.layout.**Baseline**- org.jdesktop.layout.**GroupLayout** (implements java.awt.LayoutManager2)- org.jdesktop.layout.**GroupLayout.Group**
        - org.jdesktop.layout.**GroupLayout.ParallelGroup**- org.jdesktop.layout.**GroupLayout.SequentialGroup**
        - org.jdesktop.layout.**LayoutStyle**

---


|  |  |  |
| --- | --- | --- |
| **Package** \| Class \| Use \| **Tree** \| **Deprecated** \| **Index** \| **Help** | |  |
| PREV   NEXT | **FRAMES**    **NO FRAMES**     **All Classes** |  |


---
